# Interaction of p53 with the Δ133p53α and Δ160p53α isoforms regulates p53 conformation and transcriptional activity

**DOI:** 10.1038/s41419-024-07213-4

**Published:** 2024-11-19

**Authors:** Fanny Tomas, Pierre Roux, Véronique Gire

**Affiliations:** CRBM, University of Montpellier, CNRS, Montpellier, France

**Keywords:** Mechanisms of disease, Tumour-suppressor proteins

## Abstract

The *TP53* gene encodes p53, a transcription factor involved in tumor suppression. However, *TP53* also encodes other protein isoforms, some of which can disrupt the tumor suppressor functions of p53 even in the absence of *TP53* mutations. In particular, elevated levels of the Δ133*TP53* mRNA are detected in many cancer types and can be associated with poorer disease-free survival. We investigated the mechanisms of action of the two proteins translated from the Δ133*TP53* mRNA: the Δ133p53α and Δ160p53α isoforms, both of which retain the oligomerization domain of p53. We discovered that the Δ133p53α and Δ160p53α isoforms adopt an altered conformation compared to full-length p53, exposing the PAb240 epitope (RHSVVV), which is inaccessible to the PAb240 antibody in the functional conformation of p53 (reactive to PAb1620). The Δ133p53α and/or Δ160p53α isoforms form hetero-oligomers with p53, regulating the stability, the conformation and the transcriptional activity of the p53 hetero-oligomers. Under basal conditions, Δ133p53α and Δ160p53α, in complex with p53, prevent proteasome-dependent degradation leading to the accumulation of PAb240 reactive Δ133p53α/Δ160p53α/p53 hetero-oligomers without increasing p53 transcriptional activity. Conversely, depletion of endogenous Δ133p53α isoforms in human fibroblasts is sufficient to restore p53 transcriptional activity, towards p53-target genes involved in cell cycle arrest. In the DNA damage response (DDR), PAb240 reactive Δ133p53α/Δ160p53α/p53 hetero-oligomers are highly phosphorylated at Ser15 compared to PAb1620-reactive p53 complexes devoid of Δ133p53α and Δ160p53α. This suggests that PAb240-reactive p53 hetero-oligomers integrate DNA damage signals. Δ133p53α accumulation is a late event in the DDR that depends on p53, but not on its transcriptional activation. The formation of Δ133p53α and p53 complexes increases at later DDR stages. We propose that Δ133p53α isoforms regulate p53 conformation as part of the normal p53 biology, modulating p53 activity and thereby adapting the cellular response to the cell signals.

## Introduction

The transcription factor p53 regulates cell fate decisions in response to cellular stress [1]. Upon DNA damage, p53 is rapidly stabilized and activates the transcription of genes involved in many responses, ranging from transient cell cycle arrest and DNA repair, to terminal outcomes, such as senescence and apoptosis [2, 3]. Through these activities, p53 suppresses cancer development, by preventing the emergence and proliferation of genetically unstable cells. To maintain its proper function, its levels are tightly controlled by negative regulators [4] among which many are p53 target genes that form negative feedback loops. Disruption of p53 function, which is common in cancer cells, due to defects in its regulators or *TP53* mutations (present in over 50% of cancer types), can promote tumor development [5–7].

The human *TP53*, produces multiple isoforms through internal promoter usage and alternative splicing [8, 9]. These isoforms, including the Δ133p53 isoforms family, can disrupt p53 tumor suppressor functions even in the absence of *TP53* mutations [8]. The Δ133p53 isoforms lack the first 132 amino acids and are produced from an internal *TP53* promoter located in intron 4, with alternative C-terminal splicing of exons 9, 9β and 9γ, resulting in the α, β and γ isoforms. An alternative translation initiation site, within the Δ133*TP53* mRNA generates two isoforms, Δ133p53 and Δ160p53 [8, 9]. These isoforms are frequently overexpressed in human tumors [10, 11], particularly high-grade tumors, and are associated with increased risk of metastasis and recurrence [12, 13]. The Δ133p53α and β isoforms can promote tumor development, invasion and metastasis through p53-dependent and -independent mechanisms [10, 12, 14, 15]. However, the mechanisms involved in the biological activity of Δ133p53α and Δ160p53α isoforms are not well understood. In normal conditions, Δ133p53α activities may contribute to cell homeostasis and functions. Δ133p53α lacks part of the p53 DNA-binding domain, but retains the oligomerization domain to interact with full-length p53 [16]. For example, autophagic degradation of Δ133p53α releases p53 from inhibition, leading to the full activation of the p53-mediated senescence program [17, 18]. However, p53 inactivation by Δ133p53α does not fully explain its pro-tumorigenic effect.

We previously reported that the N-terminal truncation in Δ133p53β results in a protein with a very low structural stability and an unfolded conformation similar to that of structural p53 mutants [14]. Wild-type p53 is a flexible protein and conformationally labile protein, and hetero-oligomers of wild-type p53 with mutant p53 can lead to the adoption of a mutant conformation by wild-type p53 [19]. Regulated changes in p53 protein conformation are important for its activity. Wild-type p53 can oscillate between wild-type and mutant-like conformations in a cell cycle-dependent manner [20]. In specific conditions, for instance upon depletion of chaperones, wild-type p53 can adopt a mutant conformation, leading to mutant-like functions, such as increased cancer cell invasion [21] and gene expression pattern changes [22]. Here, we report that Δ133p53α and Δ160p53α isoforms, by forming hetero-oligomers with p53, can shift the wild-type p53 conformation towards an altered conformation, thus promoting p53 protein accumulation and impairing p53 anti-proliferative activity.

## Results

### Δ133p53α and Δ160p53α overexpression stabilizes p53 without increasing its transcriptional activity

**Fig. 1:**
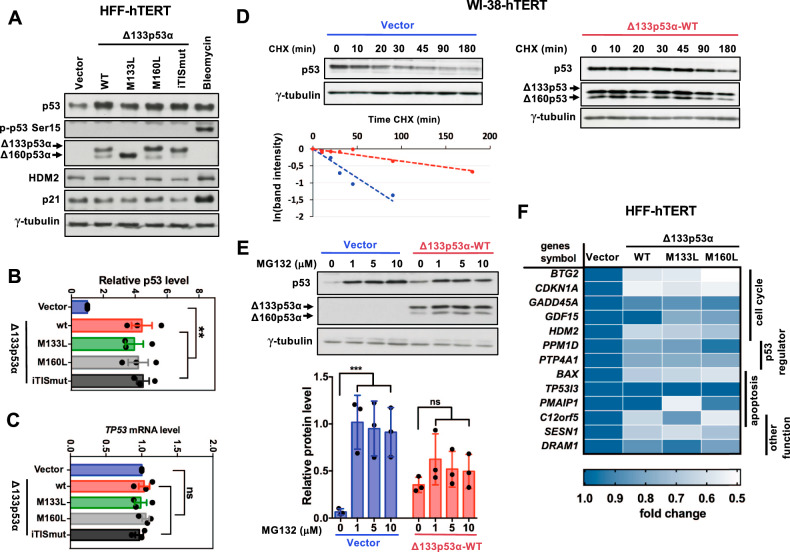
Δ133p53α and Δ160p53α overexpression promotes p53 protein stabilization without increasing its transcriptional activity. **A** p53 levels are increased in HFF-hTERT cells that overexpress wild-type (WT) Δ133p53α, the M133L, M160L or iTISmut mutants. HFF-hTERT cells were infected with retroviral vectors expressing the indicated Δ133p53 variants or empty vector (vector). Expression of p53 (DO1 antibody), phosphorylated p53 at Ser15 (p-p53 Ser15), HDM2, p21, Δ133p53α and Δ160p53α (DO11 antibody) was determined by immunoblotting at day 8 after infection. γ-tubulin, loading control. Cells incubated with bleomycin were used as a positive control for p53 stability, phosphorylation on Ser15, and activity. The Δ133*TP53* cDNA construct produces the Δ133p53α isoform (a 35kDa band) and the Δ160p53α isoform (a 32kDa band). **B** Quantification of p53 from (**A**). Band intensity was quantified with ImageJ and p53 protein levels were normalized to γ-tubulin levels. Expression is relative to control cells (vector alone). Data are the mean ± SEM (*n* = 3); **p < 0.01, one-way ANOVA with Dunnett’s multiple comparison test. **C** RT-qPCR was performed using RNA extracted from the cell cultures described in (**A**) to measure *TP53* expression (primers hybridizing within the p53 transactivation domain). *TP53* expression levels were normalized to *TBP* expression, and data were expressed as relative values. Data are the mean ± SEM (*n* = 3); ns, not significant, one-way ANOVA with Dunnett’s multiple comparison test. **D** The half-life of p53 is increased in WI-38-hTERT cells that overexpress Δ133p53α and Δ160p53α isoforms. WI-38-hTERT cells expressing vector alone (vector) or Δ133p53α-WT (Δ133p53α-WT) were incubated with cycloheximide (CHX) for the indicated times and the levels of endogenous p53 and overexpressed Δ133p53α and Δ160p53α were determined by immunoblotting. Panels are representative of three independent experiments. The graph (bottom panel) shows p53 quantification with ImageJ software in control (vector alone, blue line) and Δ133p53α-WT-expressing cells (red line). p53 levels were normalized to γ-tubulin, and then to the *t* = 0 value in control. **E** Proteasome-dependent degradation of p53 is inhibited by Δ133p53α and Δ160p53α. HFF-hTERT cells that express vector alone or Δ133p53α-WT were incubated with the proteasomal inhibitor MG132 at the indicated concentration for 6 hours, and p53 levels were determined by immunoblotting. Band intensity was quantified with Image J software and normalized to γ-tubulin levels. Data are the mean ± SEM (*n* = 3). ***p < 0.001; ns, not significant, two-way ANOVA with Dunnett’s multiple comparison test. **F** The mRNA levels of a panel of p53 target genes were determined by RT-qPCR in HFF-hTERT cells that overexpress Δ133p53α-WT (WT), Δ133p53α-M133L (M133L), or Δ133p53α-M160L (M160L) at day 8 after infection. In the heatmap, colors indicate fold-change compared with control cells (vector alone) (*n* = 3).

Mutant p53 proteins strongly accumulate in tumor cells due to increased protein stability [23, 24]. We overexpressed Δ133p53α-WT isoform in non-transformed immortalized human fibroblast cells (HFF-hTERT and WI-38-hTERT) that express functional p53 to test its effect on p53 levels. We chose non-transformed cells because some signaling pathways are altered in cancer cells and this affects the response to p53 activation [25]. As Δ133*TP53* mRNA can produce naturally two p53 isoforms, Δ133p53α (35 kDa band) and Δ160p53α (32 kDa band), we generated point mutations in Δ133p53α cDNA by site-directed mutagenesis [9]. Substitution of the methionine at codon 133 with a leucine (M133L) abolished Δ133p53α expression (35 kDa band), whereas substitution of the methionine at codon 160 with a leucine (M160L) abolished expression Δ160p53α expression (32 kDa band). Overexpression of Δ133p53α-WT, Δ133p53α-M133L or Δ133p53α-M160L increased p53 levels compared with control cells (vector alone) in all tested cell types (Fig. 1A, B; Supplementary Fig. S1A). As Δ133*TP53* mRNA contains seven internal translation initiation sites (iTIS) that could produce truncated forms of Δ133p53α protein, we generated a construct in which all seven internal methionine residues downstream of 133 were replaced by a leucine in order to express only the full length Δ133p53α. Expression of this construct, called Δ133p53α-iTISmut (iTISmut; for iTIS mutated), in HFF-hTERT cells mimicked the expression of Δ133p53α-WT (Fig. 1A, B). On the other hand, *TP53* mRNA levels were not significantly increased in cells expressing Δ133p53α-WT or its variants compared with control (Fig. 1C). The higher p53 levels may be due to increased protein stability, resulting from phosphorylation at Ser15 in response to constitutively activated DDR signaling in the presence of Δ133p53α variants. However, we could not detect p53 phosphorylated at Ser15 in cells that overexpressed Δ133p53α-WT, Δ133p53α-M133L, Δ133p53α-M160L or Δ133p53α-iTISmut, indicating that constitutive DNA damage does not contribute to p53 stabilization (Fig. 1A).

To test the influence of p53 turnover alterations on p53 stabilization in Δ133p53α-WT overexpressing WI-38-hTERT cells, we determined p53 half-life using cycloheximide to block protein synthesis. In control cells, p53 levels decreased over time, but not in Δ133p53α-WT-expressing cells (Fig. 1D). The half-life of p53 was approximately 30 minutes in control cells and 180 minutes in Δ133p53α-WT-overexpressing cells (Fig. 1D). HDM2, a transcriptional target of p53, regulates p53 levels mainly by facilitating its ubiquitination and proteasomal degradation [26]. To investigate whether impaired HDM2 priming for proteasomal degradation contributed to p53 stabilization, we incubated Δ133p53α-WT-overexpressing WI-38-hTERT cells with increasing concentrations of the proteasome inhibitor MG132 and then assessed p53 levels by western blotting. In the presence of MG132, p53 levels were significantly stabilized in control cells (by approximately 20-fold compared with untreated cells) and also in Δ133p53α-WT-overexpressing cells, although to a lesser extent (only by 2-fold) (Fig. 1E). Importantly, Δ133p53α and Δ160p53α isoforms were degraded by the proteasome, because their stability increased upon incubation with MG132 (Fig. 1E). These data suggest that Δ133p53α-WT co-expression inhibits the proteasome-dependent degradation of p53, resulting in p53 stabilization.

Next, we tested whether stabilized p53 was transcriptionally active in cells that overexpress Δ133p53α and Δ160p53α isoforms. The levels of p21 and HDM2 proteins, two known p53 transcriptional targets, were not increased compared with control cells despite p53 stabilization (Fig. 1A). As a positive control, their expression in parental HFF-hTERT cells was induced by incubation with bleomycin, a DNA damage agent that also stabilizes p53. RT-qPCR confirmed that the basal expression of many p53 target genes was differentially reduced in cells overexpressing Δ133p53α-WT, Δ133p53α-M133L, Δ133p53α-M160L or Δ133p53α-iTISmut compared to control cells (Fig. 1F; Supplementary Fig. S1B). Similarly, in immortalized human mammary epithelial cells (HMEC-hTERT), p53 levels increased while p53 transcriptional activity was reduced upon overexpression of Δ133p53α-WT, Δ133p53α-M133L, or Δ133p53α-M160L compared to the control (vector) (Supplementary Fig. S1C). Overall, these results demonstrate that in basal conditions overexpression of Δ133p53α-WT, Δ133p53α-M133L, Δ133p53α-M160L or Δ133p53α-iTISmut stabilizes p53, without increasing its transcriptional activity.

### Δ133p53α-WT overexpression prevents growth suppressive activity of p53

**Fig. 2:**
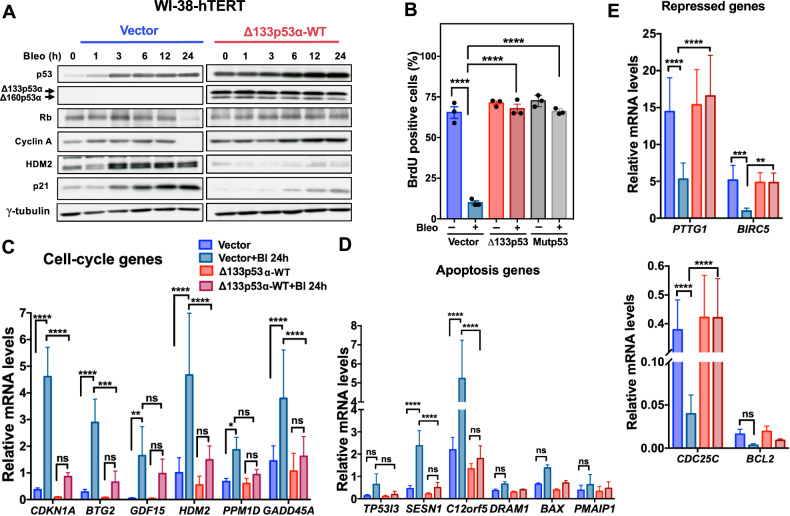
Δ133p53α-WT overexpression prevents growth suppressive activity of p53. **A** Δ133p53α-WT-expressing WI-38-hTERT cells and control cells (vector alone) were incubated or not with bleomycin (Bleo) for the indicated time and the expression of p53, pRb, cyclin A, HDM2, p21, Δ133p53α and Δ160p53α was determined by immunoblotting. γ-tubulin, loading control. Data are representative of three independent experiments. **B** Proliferation of WI-38-hTERT cells that express Δ133p53α-WT (Δ133p53), the mutant p53R175H (mutp53) or vector alone (control) measured using the BrdU incorporation assay. The graph shows the percentage of BrdU-positive cells 24 hours after incubation with bleomycin. Data are the mean ± SEM (*n* = 3); ****p < 0.0001, one-way ANOVA with Dunnett’s multiple comparison test. **C**–**E** Expression (RT-qPCR) of p53 target genes in Δ133p53α-WT-expressing WI-38-hTERT cells and control cells (vector) after incubation or not with bleomycin (Bl) as in (**A**) for 24 hours: genes induced by p53 and involved in cell cycle arrest (**C**) or apoptosis (**D**), and genes repressed by p53 (**E**). Expression levels are relative to those in untreated control cells (vector). Data are the mean ± SEM (*n* = 4); **p < 0.001, ***p < 0.001, ****p < 0.0001, ns, not significant, two-way ANOVA with Tukey’s multiple comparison test.

We next investigated the consequences of p53 stabilization by Δ133p53α-WT on p53-dependent cell cycle arrest in response to DNA damage. We incubated WI-38-hTERT cells with bleomycin and harvested them at different time points. p53 was rapidly stabilized in control cells and in Δ133p53α-WT-expressing cells after bleomycin addition (Fig. 2A). Conversely, p21 and of HDM2 were very weakly induced in Δ133p53α-WT-expressing cells compared with control cells where they were robustly induced. After 24 hours, pRb and cyclin A levels were reduced in control cells, but not in Δ133p53α-WT-expressing cells (Fig. 2A). In Δ133p53α-WT-expressing cells, like in cells expressing the p53-R175H structural mutant, most cells were BrdU-positive (Fig. 2B), indicating their ability to override the cell cycle arrest. Activation of key ATM-dependent signaling transducers and p53 phosphorylation at Ser15 were not different in Δ133p53α-WT-expressing and control cells (Supplementary Fig. S2A), suggesting that other mechanisms are involved in the reduced p53 transcriptional activity.

To evaluate Δ133p53α and Δ160p53α effect on p53 transcriptional activity, we performed RT-qPCR to measure in WI-38-hTERT cells the expression of twelve direct p53 targets involved in p53 autoregulation, cell cycle arrest, apoptosis, and metabolism. In control cells, incubation with bleomycin significantly induced the expression of eight target genes (Fig. 2C, D), mainly involved in cell cycle arrest (e.g. *CDKN1A*, *GDF15* and *BTG2)*, whereas pro-apoptotic target genes (e.g. *TP53i3*, *BAX* and *PMAIP1*) were not induced. This is consistent with the notion that p53 activation after DNA damage causes cell cycle arrest, but not apoptosis in normal fibroblasts [27]. Conversely, overexpression of Δ133p53α-WT decreased target gene induction and attenuated the repression of *CDC25C, PTTG1*, and *BIRC5* (targets of p53 transcriptional repression) upon bleomycin treatment (Fig. 2C–E). Moreover, p53 transcriptional response was defective in Δ133p53α-M133L and Δ133p53α-M160L-overexpressing HFF-hTERT cells, compared with control cells (Supplementary Fig. S2B). The negative activity of Δ133p53α-WT against p53 was confirmed by the negligible changes observed in MDAH041 (p53-null) fibroblasts (Supplementary Fig. S2C). These results show that Δ133p53α-WT overexpression in normal fibroblasts impairs p53 transcriptional activity and its ability to regulate cell cycle arrest in response to DNA damage.

### Δ133p53α-WT impairs p53 activity by binding to its oligomerization domain

**Fig. 3:**
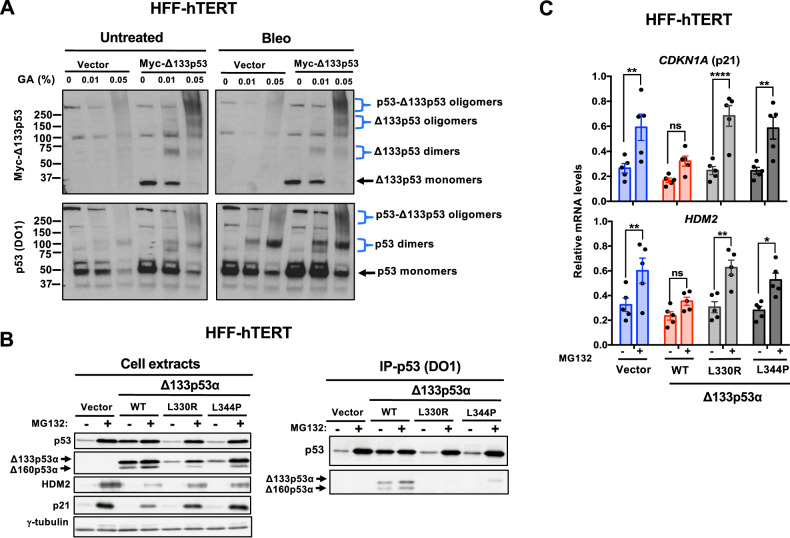
Δ133p53α impairs p53 activity by binding to its oligomerization domain. **A** Higher order oligomers in HFF-hTERT cells that express Myc-tagged-Δ133p53α or not (vector). Cells were incubated or not (Untreated) with bleomycin (Bleo) for 6 hours and then cross-linked with glutaraldehyde (GA), followed by separation. Western blot analysis was performed with an anti-Myc antibody to detect Myc-tagged-Δ133p53α (top panels), followed by the DO1 antibody to detect p53 (bottom panels). Images are representative of three independent experiments. **B** Δ133p53α/Δ160p53α oligomerization domain mutants cannot form a complex with p53. HFF-hTERT cells that express Δ133p53α-WT (WT), the oligomerization mutants L344P or L330R, or vector alone (control) were incubated with or without MG132 (1 µM) for 6 hours. Binding of the Δ133p53α and Δ160p53α isoforms to p53 was determined by immunoprecipitation using the DO1 antibody that recognizes only full-length p53. Lysates (left panels) used for the immunoprecipitation and immunoprecipitates (right panels) were analyzed by western blotting with antibodies against p53 (DO1) and Δ133p53α and Δ160p53α (DO11). The expression levels of p21 and HDM2 were determined by immunoblotting. γ-tubulin, loading control. **C** Δ133p53α/Δ160p53α and p53 complex formation is required for Δ133p53α-WT effect on p53 target gene expression. RT-qPCR was performed using RNA extracted from the cell cultures described in (**B**) to measure *CDKN1A* and *HDM2* expression. mRNA levels were normalized to *TBP* levels. Data are the mean ± SEM (*n* = 5); *p < 0.05, **p < 0.01, ****p < 0.0001, ns, not significant, two-way ANOVA with Sidak’s multiple comparison test.

As p53 functions as a tetramer [28] and Δ133p53α isoforms share the oligomerization domain with p53 [8], Δ133p53α isoforms may impair p53 transcriptional response by forming transcriptionally defective oligomeric complexes with p53. To investigate whether Δ133p53α-WT forms hetero-oligomers with endogenous p53, we cross-linked with glutaraldehyde (GA) protein extracts from HFF-hTERT cells that express Myc-tagged Δ133p53α-WT or vector alone and incubated or not bleomycin. The DO1 antibody, which recognizes p53 but not the Δ133p53α isoforms, detected one band at >250 kDa that corresponded to p53 oligomers, one band at 100 kDa that corresponded to p53 dimers, and another band at 50 kDa that corresponded to p53 monomers (Fig. 3A). The anti-Myc antibody showed that Δ133p53α formed oligomers with p53 in samples incubated with 0.05% GA. Both antibodies detected bands corresponding to p53 oligomers, p53/Δ133p53α oligomers, p53 dimers, and Δ133p53α dimers only in GA-treated cell extracts. Only p53 monomers, but not Δ133p53α monomers, were present in cell extracts incubated with 0.05% GA (Fig. 3A). Thus, Δ133p53α oligomerizes with p53 and forms oligomers with activated p53 (bleomycin condition).

As Δ133p53α and Δ160p53α isoforms interact with p53 through their oligomerization domain, we generated Δ133p53α-WT variants (Δ133p53α-L344P and Δ133p53α-L330R) carrying a single point mutation in the oligomerization domain [29, 30]. We found that in HFF-hTERT cells, the transcript levels of Δ133p53α-L344P and Δ133p53α-L330R were similar to those of Δ133p53α-WT, but the protein levels were much lower (Supplementary Fig. S3A, B). To increase the amount of oligomeric variant proteins, we incubated HFF-hTERT cells that express these variants with MG132 to stabilize their protein level (Fig. 3B, left panels) and then performed p53 immunoprecipitation, using the DO1 antibody. Δ133p53α-WT, but not Δ133p53α-L344P and Δ133p53α-L330R, co-immunoprecipitated with endogenous p53 (Fig. 3B, right panels). We obtained, similar results using Δ133p53α-L344P and Δ133p53α-L330R-expressing HFF-hTERT cells not incubated with MG132 after normalizing the input material to that of Δ133p53α-WT (Supplementary Fig. 3C, D). These results suggest that L330 and L344 in Δ133p53α-WT oligomerization domain are important for the formation of p53 and Δ133p53α-WT complexes.

To investigate the effect of the oligomerization domain mutations on Δ133p53α-WT function, we analyzed the expression of p21 (*CDKN1A*) and *HDM2* by RT-qPCR and western blotting in HFF-hTERT cells that express the oligomeric mutants after incubation with MG132 to stabilize their protein levels and activate p53 (Fig. 3B, left panels). The oligomeric mutants Δ133p53α-L344P and Δ133p53α-L330R, which cannot form a complex with endogenous p53 (Fig. 3B, right panels), showed no effect on p21 and HDM2 mRNA and protein expression (Fig. 3B, C). Altogether, these results suggest that Δ133p53α-WT ability to impair p53-responsive gene expression requires oligomerization with p53.

### The presence of Δ133p53α-WT in p53 hetero-oligomer complexes causes a conformational change in p53

**Fig. 4:**
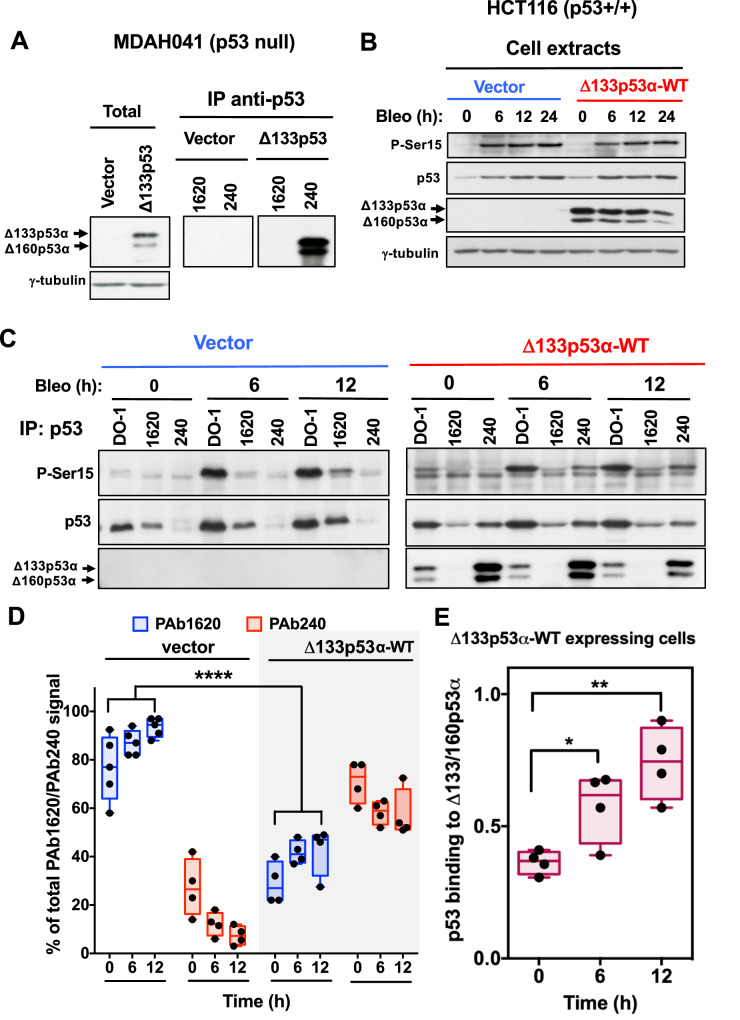
The presence of Δ133p53α and Δ160p53α in p53 hetero-oligomer complexes leads to a conformational change in p53. **A** Δ133p53α and Δ160p53α conformation was analyzed using two conformation-specific monoclonal antibodies: PAb1620 and PAb240. Lysates from MDAH041 fibroblasts that express or not Δ133p53α-WT (Δ133p53) were immunoprecipitated with PAb1620 or PAb240. Δ133p53α and Δ160p53α in total cell lysates (left panels) and immunoprecipitates (right panels) were detected by western blotting with the DO11 antibody. γ-tubulin, loading control. Images are representative of three independent experiments. **B** HCT116 cells that overexpress Δ133p53α-WT or not (vector) were incubated with bleomycin (Bleo) for the indicated time to induce p53. p53 phosphorylated at Ser15 (P-Ser15), p53 (DO1) and Δ133p53α and Δ160p53α (DO11) were detected by Western blotting. γ-tubulin, loading control. Images are representative of four independent experiments. **C** Lysates from the experiment shown in (**B**) were immunoprecipitated with PAb1620 or PAb240: p53 phosphorylated at Ser15 (P-ser15), p53 (DO1) and Δ133p53α and Δ160p53α (DO11) in the immunoprecipitates were detected by western blotting. **D** Conformation change of p53 in the immunoprecipitates shown in (**C**) was calculated as the ratio of the p53 band intensities precipitated by PAb1620 to those precipitated by PAb240. Box plots show the data range (whiskers) and median (middle line) (*n* = 4); ****p < 0.0001, two-way ANOVA with Tukey’s multiple comparison test. **E** Quantification of p53 signals using ImageJ software, normalized to the Δ133p53α and Δ160p53α signals in the immunoprecipitates from the experiments shown in (**C**). Box plots show the data range (whiskers) and median (middle line) (*n* = 4); *p < 0.05, **p < 0.01, one-way ANOVA with Dunnett’s multiple comparison test.

In Δ133p53α-WT-overexpressing cells, p53 stability is increased and its transcriptional activity is impaired, as observed for mutant p53 found in cancer cells [31]. p53 is a conformationally labile protein that can be inactivated by mutations altering its functional conformation. Changes in p53 conformation can be detected using two antibodies: i) PAb1620 recognizes a domain only present in the correctly folded protein (functional wild-type p53) [32] and ii) PAb240 recognizes a hidden epitope RHSVVV (residues 213-218) only exposed after denaturation of the protein or in specific type of p53 mutants with altered folding such as p53-R175H [19, 33]. Immunoprecipitation assays in Δ133p53α-WT-expressing MDAH041 (p53-null) fibroblasts indicated that the Δ133p53α and Δ160p53α isoforms could only be precipitated by PAb240 (Fig. 4A), suggesting that deletion of p53 N-terminal portion affects protein conformation and exposes normally masked epitopes.

We next analyzed the endogenous p53 conformation in Δ133p53α-WT-expressing cells. However, it was difficult to estimate the amount of p53 in the PAb240 reactive conformation due to co-immunoprecipitation with Δ133p53α and Δ160p53α. We used PAb1620, which does not recognize Δ133p53α and Δ160p53α (Fig. 4A), to determine whether Δ133p53α-WT affected p53 conformation in HCT116 cells in which p53 was stabilized and activated by incubation with bleomycin (Fig. 4B). In untreated control cells (vector), p53 was predominantly in the functional conformation (Fig. 4C, D). Conversely, in Δ133p53α-WT-expressing cells, the amount of p53 in the functional conformation decreased. Bleomycin treatment increased the amount of p53 recognized by PAb1620 in control cells but not in Δ133p53α-WT-expressing cells (Fig. 4C, D). PAb1620-bound p53 was devoid of Δ133p53α and Δ160p53α in Δ133p53-expressing cells (Fig. 4C). PAb240-bound p53 was highly phosphorylated at Ser15, suggesting that Δ133p53α-WT regulate p53 activity by binding Ser15 activated p53 (Fig. 4C). Bleomycin also decreased Δ133p53α and Δ160p53α levels after 24 hours (Fig. 4B). Quantification of p53 in PAb240 immunoprecipitates showed an increase in p53 bound to Δ133p53α-WT after bleomycin addition (Fig. 4E). These results suggest that Δ133p53α-WT in p53 hetero-oligomer complexes leads to a p53 conformational change. This may explain how Δ133p53α-WT affects the activity of p53 hetero-oligomers.

### Disruption of the Δ133p53α-WT-p53 interaction is sufficient to activate p53 transcriptional targets and arrest the cell cycle in normal cells

**Fig. 5:**
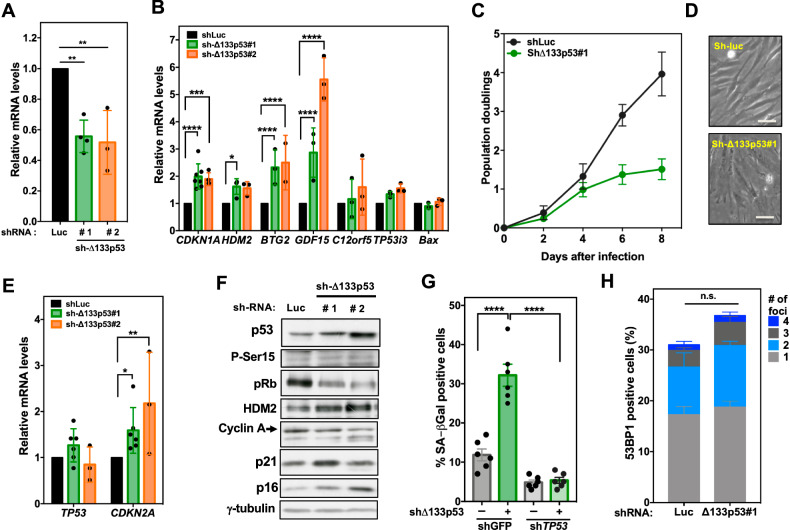
Disruption of the Δ133p53α and p53 interaction is sufficient for the activation of p53 transcriptional targets and cell cycle arrest in normal cells. **A** Confirmation of Δ133*TP53* silencing by RT-qPCR. Proliferating HFF cells were infected with a control retroviral shRNA against firefly luciferase (sh-*Luc*) or two Δ133p53 shRNAs (Δ133p53#1 and Δ133p53#2), and Δ133*TP53* expression was determined by RT-qPCR at day 8 post infection. Δ133*TP53* expression was normalized to *TBP* expression and data were expressed as relative values. Data are the mean ± SEM (*n* = 3); *p < 0.05, ***p < 0.001, ****p < 0.0001, one-way ANOVA with Dunnett’s multiple comparison test. **B** Expression (RT-qPCR) of p53 target genes in the cells shown in (**A**). Data are mean ± SEM (*n* = 3); *p < 0.05, ***p < 0.001, ****p < 0.0001, two-way ANOVA with Dunnett’s multiple comparison test. **C** HFF cells were infected with the Δ133p53#1 shRNA or sh*Luc* as in (**A**), and proliferation was assessed by counting the cell numbers at the indicated days post-infection. Data are representative of three independent experiments. **D** Representative images of HFF cells that express Δ133p53#1 shRNA or sh*Luc* at day 8 post-infection. Scale bar, 50 µm. Images are representative of three independent experiments. **E** Expression of *TP53* and *CDKN2A* was determined by RT-qPCR in HFF cells at day 8 post-infection. Data are the mean ± SEM (*n* = 6 for sh-Δ133p53#1 and *n* = 3 for Δ133p53#2); *p < 0.05, **p < 0.01, one-way ANOVA with Dunnett’s multiple comparison test. **F** HFF cells were infected as described in (**A**) and the levels of p53, phosphorylated p53 at Ser15 (P-Ser15), pRb, cyclin A (proliferative markers), HDM2, p21, p16 (senescent cell markers) and γ-tubulin (loading control) were determined by western blotting. Images are representative of three independent experiments. **G** HFF cells that express Δ133p53#1 shRNA or control sh*Luc* were infected with a retroviral construct expressing an shRNA against p53 (sh*TP53*) or against GFP (shcontrol; control) and stained for SA-β-Gal activity at day 12 after infection. SA-β-Gal-positive cells were counted relative to the total number of cells. Data are the mean ± SEM (*n* = 6); ****p < 0.0001, one-way ANOVA with Tukey’s multiple comparison test. **H** HFF cells were infected with Δ133p53#1 shRNA or sh*Luc* as in (**A**) and 53BP1 foci were detected by immunofluorescence analysis. The percentage of cells with 53BP1 foci and their number per nucleus were calculated. Data are the mean ± SEM (*n* = 3); (random fields, > 100 cells per condition); ns, not significant, two-way ANOVA with Sidak’s multiple comparison test.

Our results suggest that Δ133p53α-WT regulates the transcriptional activity of the p53 hetero-oligomers. To determine whether Δ133p53 expression is sufficient to cause this phenomenon, we silenced endogenous Δ133*TP53* mRNA in fibroblasts using two shRNAs (Sh-Δ133p53-1 and Sh-Δ133p53-2) and then examined p53 activity. Depletion of endogenous Δ133*TP53* mRNA in normal fibroblasts increased p53 transcriptional activity compared with control cells (sh-Luc) (Fig. 5A, B). Specifically, many p53 target genes involved in cell cycle arrest were upregulated, but not pro-apoptotic target genes (*TP53i3*, *BAX*). This resulted in cell growth arrest within 7 days (Fig. 5C). Moreover, the cells acquired an enlarged, flattened morphology (Fig. 5D). Compared with control, p16 mRNA and protein levels were increased (Fig. 5E, F) and also senescence-associated-beta-galactosidase (SA-β-Gal) activity (Fig. 5G). Knockdown of both Δ133*TP53* and *TP53* confirmed that p53 activation is required for senescence induction caused by Δ133*TP53* knockdown (Fig. 5G).

As DNA damage is the main p53 activator, we analyzed DDR components after Δ133*TP53* silencing. Increased p53 stability was not accompanied by p53 phosphorylation at Ser15 (Fig. 5F) or induction of 53BP1 foci (a DNA damage marker) (Fig. 5H), suggesting that the growth arrest induced by Δ133*TP53* silencing was not triggered by DDR activation. Although Δ133*TP53* silencing induced senescence (SA-β-Gal activity and p16 upregulation (Fig. 5E–G)), the expression of senescence-associated secretory phenotype (SASP) genes was not altered at day 20 post-infection with sh-Δ133p53 (Supplementary Fig. S4). These results demonstrate that Δ133*TP53* silencing enhances p53 activity without causing DNA damage or SASP induction. Thus, in normal conditions, Δ133*TP53* mRNA expression suppresses p53 activity to facilitate cell proliferation.

### Δ133p53α levels increase late after DNA damage

**Fig. 6:**
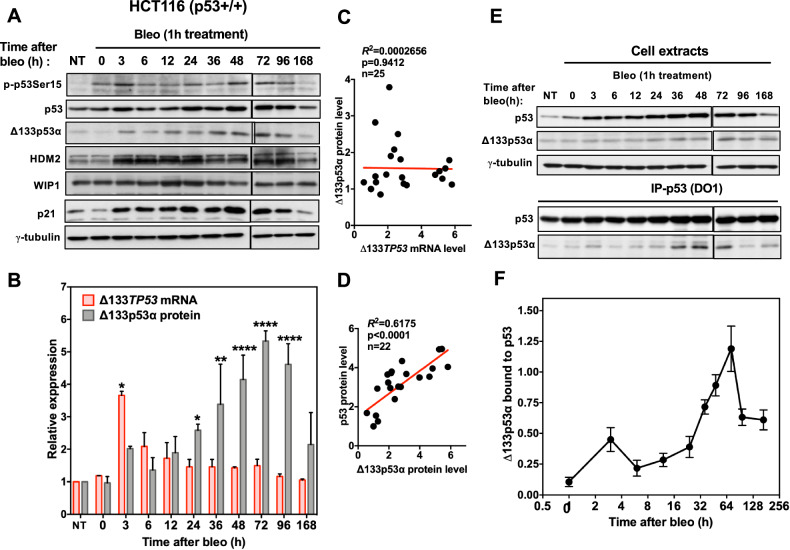
Endogenous Δ133p53α levels increase late after DNA damage. **A** Δ133p53α expression in HCT116 cells after induction of DNA damage by bleomycin. Cells were incubated with bleomycin (bleo) or not (NT) for 1 hour, released and harvested at different time points. Expression of p53, p53 phosphorylated on serine 15 (p-p53Ser15) and its targets Δ133p53α, HDM2, WIP1 and p21 was assessed by western blotting. γ-tubulin, loading control. Images are representative of three independent experiments. **B** Quantification of Δ133p53 α expression from (**A**). Δ133*TP5*3 mRNA and Δ133p53α protein levels were determined by RT-qPCR and immunoblotting, respectively. Expression is relative to control (NT) HCT116 cells. Data are the mean ± SEM (*n* = 3); *p < 0.05, **p < 0.01, ****p < 0.0001 (pairwise comparisons with the NT cells). **C** Correlation between Δ133p53α protein and Δ133*TP5*3 mRNA levels in the experiments shown in (**A**, **B**). Red line, linear regression. **D** Correlation between Δ133p53α protein and p53 protein levels in the experiments shown in (**A**). Red line, linear regression. **E** Interaction of endogenous Δ133p53α and p53 in HCT116 cells after bleomycin-induced DNA damage. Cells were incubated with bleomycin or not (NT), released and collected at the indicated times. Lysates were immunoprecipitated with the anti-p53 antibody (DO1). Lysates (top panels) used for immunoprecipitation experiments and immunoprecipitates (bottom panels) were analyzed by western blotting with antibodies against p53 (DO1) and Δ133p53α (DO11). γ-tubulin, loading control. Images are representative of three independent experiments. **F** Quantification of Δ133p53α signals with ImageJ, normalized to p53 signals in the immunoprecipitates from the experiments shown in (**E**). Data are the mean ± SEM (*n* = 3).

As Δ133*TP53* is a p53 target gene [34, 35], many stress signals might have the paradoxical effect of simultaneously increasing p53 abundance and Δ133p53α protein expression. To investigate the timing of Δ133p53α accumulation in response to DNA damage, we used HCT116 colon cancer cells that express p53 and Δ133p53α (Fig. 6A). We could not measure Δ160p53α protein level. We incubated cells with bleomycin for 1 hour and then cultured them for 7 days. During the early DDR (up to 12 hours), the levels of total and phosphorylated p53 (Ser15), p21 and HDM2 increased, but not Δ133p53α (Fig. 6A, B; Supplementary Fig. S5A). Δ133p53α started to increase at 24 hours and continued to increase, peaking at 72 hours (Fig. 6B; Supplementary Fig. S5A). Conversely, Δ133*TP53* mRNA expression increased at 3 hours after treatment and remained stable thereafter (Fig. 6B). Δ133p53α levels did not correlate with Δ133*TP53* mRNA expression, but only with p53 accumulation (Fig. 6C, D), whereas p21 level correlated with *CDKN1A* mRNA level (Supplementary Fig. S5C). At 96 hours, when p53 target gene activation began to decline, Δ133p53α stability was associated with a reduction in p53 transcriptional activity, although p53 remained phosphorylated at Ser15 (Fig. 1A; Supplementary Fig. S5B). Late Δ133p53α accumulation required p53 because Δ133p53α expression increase was prevented in HCT116 p53^-/-^ cells (Supplementary Fig. S5D). These results demonstrate that Δ133p53α accumulation is a late event and is not consistent with an acute short-term DDR.

As we detected stable Δ133p53α-WT and p53 complexes in undamaged cells (Fig. 3A, B), we asked whether the two endogenous proteins interacted in response to DNA damage after incubation with bleomycin. Their interaction was detectable already at 3 hours post-incubation and peaked between 48 and 72 hours (Fig. 6E, F). These results indicate that the formation of Δ133p53α and p53 complexes is increased at later time points during the DDR.

## Discussion

Our findings reinforce the concept that p53, a flexible protein regulated by its conformation, can adopt different functional states depending on the context [20, 21, 36]. Correct folding of p53 is critical for binding to specific DNA sequences to activate target genes. Mutations in p53 often alter its conformation and cause unfolding of the protein, resulting in the loss of its binding ability to some p53-response elements [37]. Here, we found that the altered conformation of Δ133p53α and/or Δ160p53α can induce wild-type p53 to adopt a different conformation in the hetero-oligomer complex, reducing p53’s ability to activate target genes involved in cell cycle arrest following DNA damage. This reveals that regulation of p53 conformation by Δ133p53α and/or Δ160p53α is an important and novel mechanism for controlling p53 transcriptional responses. Our results support the understanding that the primary function of Δ133p53α in relation to p53 would be a negative regulator interacting with p53 in a regulatory feedback loop [17, 34]. Moreover, Δ133p53α reduces the transcription of canonical p53 target genes during the DDR, suggesting that inhibition of p53 transcriptional activation may not be restricted to a subset of genes involved in senescence [16, 17].

Our findings imply that regulation of p53 conformation may be part of its normal biology. Active regulation of conformation due to the interaction between p53 and Δ133p53α and/or Δ160p53α can affect p53 activity, even in the absence of mutations in the DNA binding domain of p53. Therefore, the appropriate balance between p53 and Δ133p53α and Δ160p53α must be preserved to regulate p53 functions because their relative levels determine the biological outcome. In proliferating human fibroblasts, our data indicate that Δ133p53α inhibits p53 anti-proliferative activity. Reducing the number of Δ133p53α/p53 hetero-oligomers (by silencing Δ133*TP53* mRNA or expression of oligomerization-defective mutants) relieves p53 inhibition, restores its active conformation that enables the transactivation of a subset of p53 inducible genes such as the ones involved in cell cycle arrest (Fig. 5). Conversely, an excess of Δ133p53α and Δ160p53α (relative to p53) impairs the canonical p53-driven response to the DNA damaging agent bleomycin (Fig. 2). This altered regulation depends on the Δ133p53α/Δ160p53α/p53 interaction, because this effect is impaired by the oligomerization-defective mutants Δ133p53α-L344P and Δ133p53α-L330R (Fig. 3). The reduced p53 activity in Δ133p53α-WT-overexpressing cells may be due to Δ133p53α/Δ160p53α incorporation into hetero-tetramers that change p53 conformation to an inactive conformation (Fig. 4), thus preventing the binding of active p53 (phosphorylated at Ser15) to the promoters of relevant target genes and/or the interaction with cofactors [16]. Alternatively, high Δ133p53α and Δ160p53α levels can generate misfolded p53 aggregates [14] because these isoforms stabilize p53 and downregulates HDM2 (Fig. 1), further impairing p53 transcriptional function. We cannot completely rule out that the Δ133p53α/Δ160p53α/p53 hetero-oligomers may function as DNA-binding transcriptional regulators in a promoter context-dependent manner and/or at another set of genes in human fibroblasts [10, 34].

As the regulation of p53 conformation by Δ133p53α may be part of the normal p53 biology, Δ133p53α level should be tightly controlled. Δ133*TP53* is a direct transcriptional target of p53 [34, 35] and is weakly expressed in proliferating cells. In HCT116 cells after bleomycin-induced DNA damage, Δ133*TP53* mRNA expression was slightly increased in the early DDR and required p53 (Fig. 6B and Supplementary Fig. S5D). In the late DDR, Δ133p53α protein accumulation correlated with p53 levels and persistent DNA damage, but not with its mRNA level (Fig. 6A–D). Our data suggest that DNA damage signaling may regulate Δ133p53α levels, possibly through post-translational stabilization, and that Δ133p53α upregulation may be involved in the late DDR. Consequently, the Δ133p53α to p53 ratio should increase during the stress adaptive response when p53 levels are reduced due to less damage. Conversely, in response to acute DNA damage, p53, but not Δ133p53α, is stabilized, leading to p53-dependent cell cycle arrest. In agreement, a previous study linked Δ133p53α degradation *via* chaperone-assisted selective autophagy to full p53 activation during replicative senescence entry [18].

Δ133p53α and Δ160p53α role in p53 conformational shift is evident in conditions of DNA damage (Fig. 4). This raises the question of why it is advantageous for wild-type p53 to adopt different conformations *via* its transcriptional target Δ133p53α. High p53 activity triggers a terminal and irreversible response, leading to cellular senescence or apoptosis. The timely execution of these responses is an important feature of p53 function. The direct correlation between DNA damage and p53 activity can be detrimental to cell survival. Formation of p53 oligomers devoid of Δ133p53α or Δ160p53α may activate apoptosis or senescence too quickly, leaving insufficient time for damage repair and cell survival. The formation of p53/Δ133p53α and/or Δ160p53α hetero-oligomers might act as a ‘brake’ on the formation of transcriptionally active p53 oligomers (such as homo-tetramers), protecting cells from premature commitment to senescence or cell death and allowing recovery from DNA damage.

Our study has implications for understanding how Δ133p53α and/or Δ160p53α may act in cancer biology. Normal cells may contain suboptimal and rate-limiting Δ133p53α levels. Conversely, Δ133*TP53* is strongly expressed in several cancer types, particularly those with wild-type *TP*53. Δ133*TP53* high expression can be associated with poor prognosis and survival [12, 13, 38]. We showed that Δ133p53α and Δ160p53α can directly induce p53 conformational changes, impairing its pro-apoptotic and pro-senescence transcriptional activity and functions in the normal control of cell proliferation. The conformational shift of wild-type p53 towards an inactive (PAb240 reactive) functional state when Δ133p53α and Δ160p53α expression is upregulated may contribute to its oncogenic properties. High Δ133p53α and Δ160p53α levels during cancer development might force p53 to constitutively adopt an inactive conformation (PAb240 reactive). Therefore, the activity of Δ133p53α would be the abnormal manifestation of a normal state of wild-type p53 rather than the acquisition of a completely new activity. Consequently, the development of some cancers that harbor wild-type p53 could be due to the expression of an inactive conformation phenotype. This suggests that some flexibility in the activity of the active (PAb1620 reactive) and inactive (PAb240 reactive) conformations of p53 may be highly advantageous during tumorigenesis. Consistently, an analysis of human breast tumors without *TP53* mutations revealed that some of these tumors exhibited transcriptional profiles, pathway and deregulation patterns similar to those of cancers harboring mutant *TP53*. Patients with this subset of tumors had survival rates comparable to those of patients with mutant *TP53* tumors [22].

In conclusion, we report that Δ133p53α and/or Δ160p53α alters p53 conformation, stabilizes p53, impairs p53 activation and transactivation of target anti-proliferative genes. Our study suggests that the Δ133p53α to p53 ratio could be a valuable biomarker in tumors that express wild-type p53. Targeting Δ133p53α and Δ160p53α to restore the wild-type p53 conformation and function may be beneficial for patients with an inactive (PAb240 reactive) p53 conformation phenotype and poor prognosis.

## Materials and methods

### Cell culture and treatments

WI-38 (ATCC, CCL-75), HFF as described in [39], telomerase immortalized HFF (HFF-hTERT), WI-38 (WI38-hTERT), and MDAH041 cells, a p53-null fibroblast cell line derived from a patient with Li-Fraumeni syndrome kindly provided by M. Tainsky (Karmanos Cancer Institute, Detroit, MI, USA) were cultured in modified Eagle’s Medium (MEM, Gibco^TM^, ThermoFisher Scientific, Waltham, MA, USA). HCT116 and HCT116 p53^-/-^ cells, kindly provided by B. Vogelstein (Johns Hopkins University, Baltimore, MD USA), were cultured in McCoy’s 5A medium (Sigma-Aldrich, Merk Millipore, Darmstadt, Germany). All media were supplemented with 10% fetal calf serum (Eurobio Scientific, Les Ulis, France), 1% non-essential amino acids, 1 mM sodium pyruvate (Gibco^TM^), 2 mM L-glutamine (Gibco^TM^), 100 U/ml penicillin, and 10 µg/ml streptomycin (Gibco^TM^). HMEC-hTERT cells, kindly provided by J. Piette (CRBM, Montpellier, France), were grown in the Mammary Epithelial Cell Medium Kit (Lonza, Basel, Switzerland). Cells were maintained in 5% CO_2_ and ambient oxygen. Cells were tested negative from mycoplasma contamination.

BrdU incorporation to detect replicating cells was performed as reported [40] and SA-β-gal activity was assessed using the Senescence Cells Histochemical Staining Kit (Sigma-Aldrich) according to the manufacturer’s protocol.

Cells were incubated with bleomycin sulfate (Sigma-Aldrich, 10 µg/ml), cycloheximide (Sigma-Aldrich, 30 µg /ml) or MG132 (Sigma-Aldrich, 1µM).

### Retroviral plasmids and infection

The pMSCV-Hygro vector (Clontech Laboratories, now Takara Bio, San Jose, CA, USA) was used as the backbone retroviral expression vector for all Δ133p53α and Δ163p53α constructs [14]. Δ133p53α-M133L, M160L, L344P and L330R were generated by site-directed mutagenesis using the QuickChange Site-Directed Mutagenesis Kit (Stratagene, now Agilent Technologies, Santa Clara, CA, USA) according to the manufacturer’s instructions. In the Δ133p53α-iTISmut construct that produces only the full-length Δ133p53α protein, the seven internal methionine residues (ATG) downstream of 133 were replaced by a leucine (CTG) to abolish the expression of all truncated forms of Δ133p53α. This construct was generated by gene synthesis and subcloned into the pMSCV-Hygro vector (GeneArt, ThermoFisher Scientific).

To silence Δ133*TP53*, two short hairpin oligonucleotides (sh-Δ133p53#1, 5’-GGAGGTGCTTACACATGTT-3’ and sh-Δ133p53#2, 5’-CTTGTGCCCTGACTTTCAA-3’) were designed to target sequences present in the 5’ untranslated region of Δ133*TP53* but spliced out in *TP53* (intron 4). To silence all p53 isoforms, a shRNA targeting a specific sequence in the DNA binding domain of p53 was used, (sh*TP53*, 5’-GACTCCAGTGGTAATCTAC-3’). The annealed shRNA sequences were inserted into the RNAi ready pSIREN-RetroQ vector (Clontech Laboratories). All constructs were verified by DNA sequencing.

Retroviral gene experiments were described in [39]. Infected cells were selected with 100 µg/ml hygromycin and 1 µg/ml puromycin. Retroviral vectors carrying only drug resistance genes were used as controls. A pSIREN construct allowing stable expression of the shRNA targeting the firefly luciferase gene was used as control shRNA.

### Antibodies

Detailed information on the antibodies used (company names, catalogue numbers) and dilutions is provided in Supplementary Table S1.

### Cell Lysis, Immunoprecipitation and Western blotting

Cells were lysed and sonicated in 50 mM Tris-HCl pH 7.5, 150 mM NaCl, 1 mM EDTA, 0.2% NP40, 1 mM DTT supplemented with protease and phosphatase inhibitor cocktail (Sigma-Aldrich). Protein concentration was determined using the Pierce^TM^ BCA Protein Assay Kit (ThermoFisher Scientific). Cell extracts (50–80 µg of protein) were separated on SDS-polyacrylamide gels (PAGE) (Bio-Rad, Hercules, CA, USA) and transferred to Immobilon P, PVDF membranes (Millipore, Burlington, MA, USA). Immunoblotting was performed using the primary antibodies listed in Supplementary Table S1. Species-specific horseradish peroxidase-conjugated secondary antibodies were used (GE HealthCare Life Sciences, Chicago, Illinois, USA). Signals were visualized by ECL (GE HealthCare Life Sciences) and autoradiography. Band intensity was measured using ImageJ (v1.42q, http://rsb.info.nih.gov/ij). The relative protein levels were calculated after normalization to γ-tubulin.

For immunoprecipitation, cell extracts (200 µg for overexpressed proteins and 1 mg for endogenous proteins) were incubated with 2 to 10 µg of the anti-p53 antibody (DO1 (Santa-Cruz Biotechnology, Dallas, TX, USA) and the conformation-specific antibodies PAb240 and PAb1620 (provided by J-C Bourdon, University of Dundee, UK)) at 4 °C overnight, followed by 1/10 volume of Protein A/G-Sepharose beads at 4 °C for 1 h. Immunoprecipitates were separated by SDS-PAGE, and analyzed by western blotting. The mouse IgG TrueBlot ULTRA secondary antibody (Rockland, Pottstown, Pennsylvania, USA) was used to detect the immunoblotted target protein bands.

### Tetramerization assay

To analyze p53 oligomerization status, tetramerization assays using glutaraldehyde cross-linking were performed as reported [41]. Briefly, cells were lysed in 50 mM Tris-HCl pH 7.5, 150 mM NaCl, 1 mM EDTA, 0.5% NP40 supplemented with protease and phosphatase inhibitor cocktail (Sigma-Aldrich). Samples were cross-linked or not with 0.01% or 0.05% of GA at 4 °C for 30 min. Cross-linking was stopped by adding Laemmli buffer. Multimeric complexes were resolved on 4–20% gradient SDS-PAGE (Bio-Rad) followed by Western blotting with antibodies against p53 (DO1) and Myc.

### RNA extraction and reverse transcription-quantitative PCR (RT-qPCR)

Total RNA was isolated using the RNeasy Mini Kit (Qiagen, Hilden, Germany) with a DNase I treatment (Qiagen). RT was performed using 2.5 to 4 μg of total RNA, SuperScript™ III reverse transcriptase (Invitrogen) and oligo-d(T) primers. Real-time qPCR was performed using the FastStart Universal SYBR Green® Master Mix (Roche, Basel, Switzerland) on a LightCycler 480 real-time PCR instrument (Roche). The cycling program was: denaturation at 95 °C for 10 min, then 45 cycles at 95 °C for 10 s, 60 °C for 15 s, and 72 °C for 25 s. For p53 isoforms, qPCR was performed using the TaqMan® Light Cycler 480 Probes Master Mix (Roche) and 50 to 100 ng of cDNA and a LightCycler 480 real-time PCR instrument. The cycling program was: denaturation at 95 °C for 10 min, then 40 cycles of 95 °C for 30 s and 60 °C for 1 min, followed by a melting curve step. Relative mRNA levels were determined using the 2^-ΔΔCt^ method and normalized to *TBP* mRNA levels. The oligonucleotide sequences and probes are listed in Supplementary Table S2.

### Statistical analysis

Data are expressed as mean ± standard error of the mean (SEM) of a minimum of three independent experiments. Sample sizes were not predetermined with statistical methods. Statistical analysis and graph generation were performed with the GraphPad Prism software (v7.0, GraphPad Software, La Jolla, CA, USA). The used statistical tests and corrections for multiple testing are indicated in the figure legends. Statistical significance was defined as *p*-value of < 0.05.

## Supplementary information

Supplementary figures 1-5

Supplementary Table 1

Supplementary Table 2

uncropped original Western Blots

## Data Availability

All data generated or analyzed in this study are available from the corresponding author on reasonable request.
