# Supplementary material for: Interaction of p53 with the Δ133p53α and Δ160p53α isoforms regulates p53 conformation and transcriptional activity
Source: Cell Death Dis. 2024 Nov 19;15(11):845. doi: 10.1038/s41419-024-07213-4 (PMC11576908; doi:10.1038/s41419-024-07213-4)
Supplement: Supplementary file 1 — Supplementary figures 1-5 [file 41419_2024_7213_MOESM1_ESM.pdf]

Tomas_Suppl Fig 1

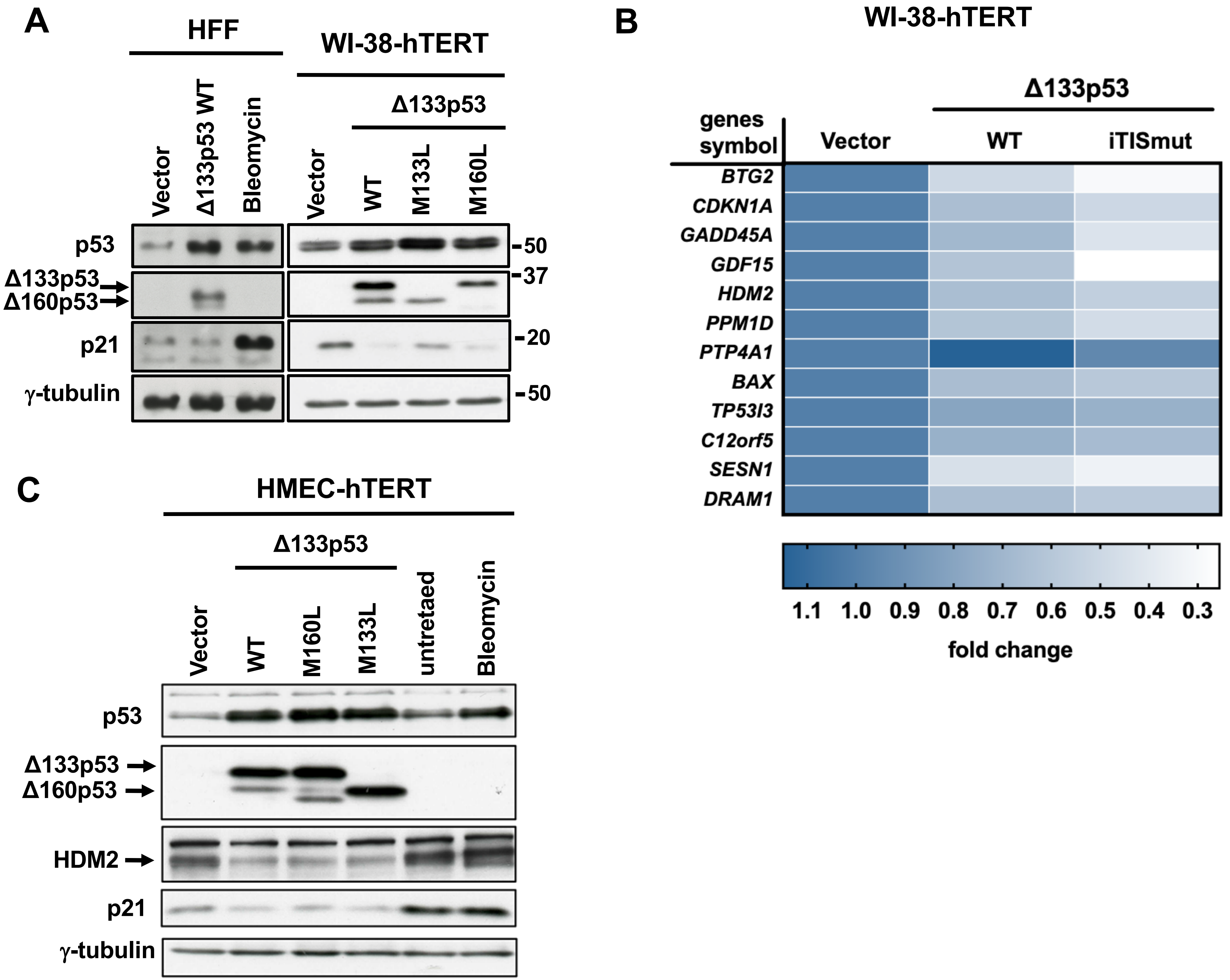

Supplementary Figure S1 (complements Figure 1). p53 is stabilized but its transcriptional activity is reduced in HFF and WI38-hTERT cells that express Δ133p53α and Δ160p53α isoforms.

(**A**) HFF and telomerase-immortalized WI-38 (WI38-hTERT) cells were infected with retroviral vectors expressing Δ133p53α-WT (WT), Δ133p53α-M133L (M133L), Δ133p53α-M160L (M160L) cDNA, or empty vector (vector). At day 8 post-infection, cells were harvested and p53 (DO1 antibody), Δ133p53α and Δ160p53α (DO11 antibody), and p21 levels were determined by immunoblotting. γ-tubulin, loading control. Bleomycin-treated cells were used as a positive control for p53 stability and activity. Images are representative of three independent experiments.

(**B**) The mRNA levels of a panel of p53 target genes were determined by RT-qPCR in WI-38-hTERT cells that overexpress Δ133p53α-WT (WT), Δ133p53α-iTISmut (iTISmut) or vector alone (vector) at day 8 post-infection. In the heatmap, colors indicate the fold-change compared with control (vector only) cells (n =3).

(**C**) Telomerase-immortalized HMEC cells (HMEC-hTERT) were infected with the Δ133p53α-WT (WT), Δ133p53α-M133L (M133L), Δ133p53α-M160L (M160L) constructs or empty vector. At day 8 post-infection, cells were harvested and endogenous p53 (DO1 antibody), Δ133p53α and Δ160p53α (DO11 antibody), p21 and HDM2 levels were detected by immunoblotting. γ-tubulin, loading control. Cells incubated with bleomycin were used as positive control for p53 stability and activity. Untreated, asynchronously growing, non-infected HMEC-hTERT cells were also used as controls.

Tomas_Suppl Fig 2

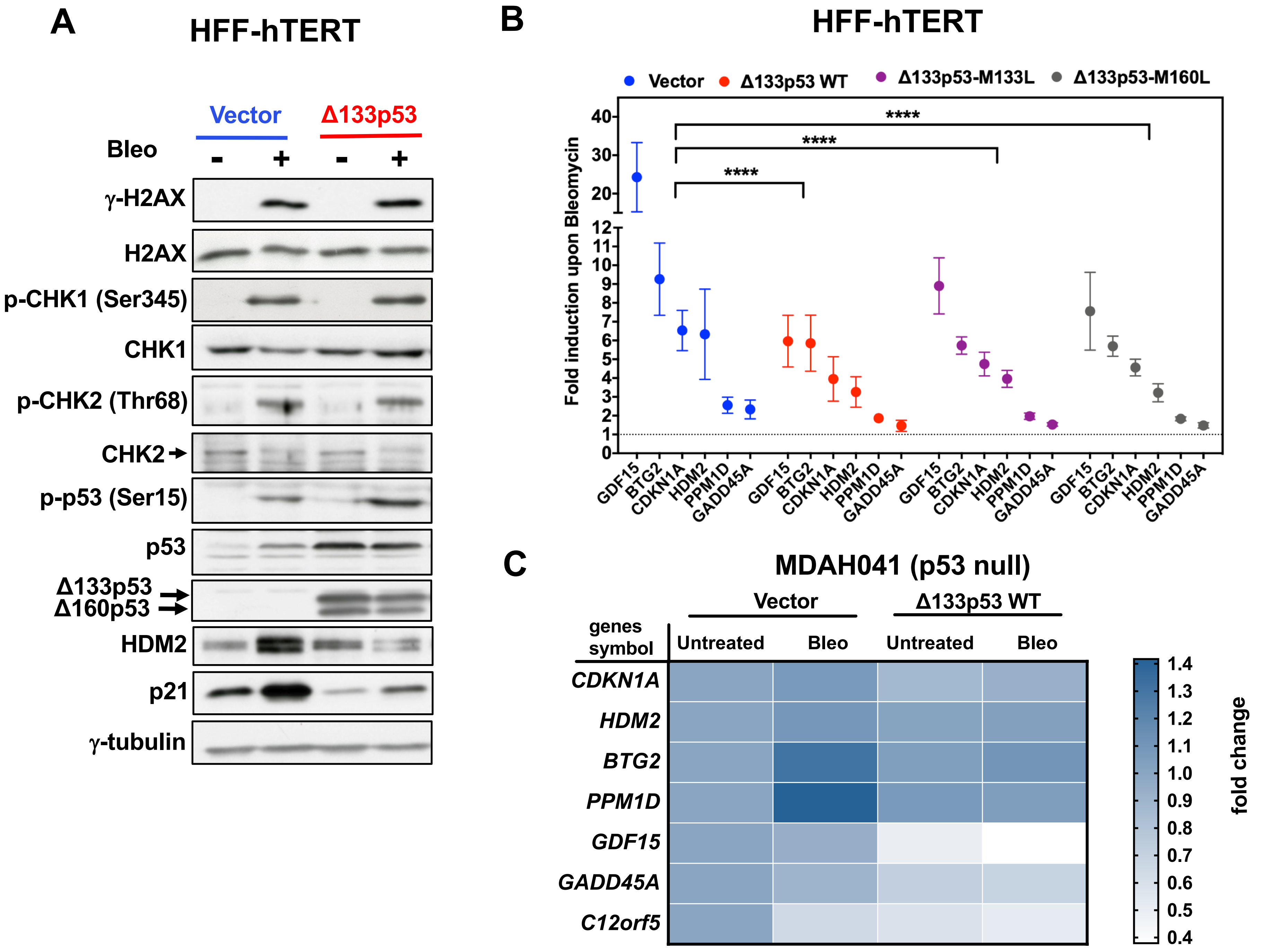

Supplementary Figure S2 (complements Figure 2). Δ133p53α and Δ160p53α controls cell proliferation by regulating p53 activity

(**A**) The p53-dependent DDR pathway upstream of p53 is functional in HFF-hTERT cells that express Δ133p53α-WT. HFF-hTERT cells that express Δ133p53α-WT or vector alone (control) were incubated or not with bleomycin for 6 hours and the levels of γ-H2AX, H2AX, CHK1 phosphorylated at Ser345 (p-Chk1 (Ser345)), CHK1, CHK2 phosphorylated at threonine 68 (p-Chk2 (Thr68)), CHK2, p53 phosphorylated at Ser15 (p-p53 (Ser15)), p53, Δ133p53α, Δ160p53α and the p53 targets HDM2 and p21 were determined by immunoblotting. γ-tubulin, loading control. Images are representative of three independent experiments.

(**B**) p53 transcriptional activity is impaired in cells that express Δ133p53α-WT, Δ133p53α-M133L, Δ133p53α-M160L constructs or vector alone (vector). RT-qPCR analysis of genes activated by p53 and involved in cell cycle arrest in HFF-hTERT cells that express Δ133p53α-WT, Δ133p53-M133L or Δ133p53-M160L and in control cells (vector only) incubated or not with bleomycin for 6 hours. mRNA expression was normalized to *TBP* values and expressed as fold induction after 6 hours of incubation relative to untreated cells. Data are the mean ± SEM ($n = 3$); ****, $p < 0.0001$, two-way ANOVA with Dunnett's multiple comparison test.

(**C**) RT-qPCR analysis of a panel of p53 target genes in MDAH041 (p53-null) fibroblasts that overexpress Δ133p53α-WT or in control cells (vector only), untreated or incubated with bleomycin for 6 hours. mRNA expression was normalized to *TBP* values and in the heatmap, colors indicate the fold-change compared with control (vector only) cells ($n = 4$).

Tomas_Suppl Fig 3

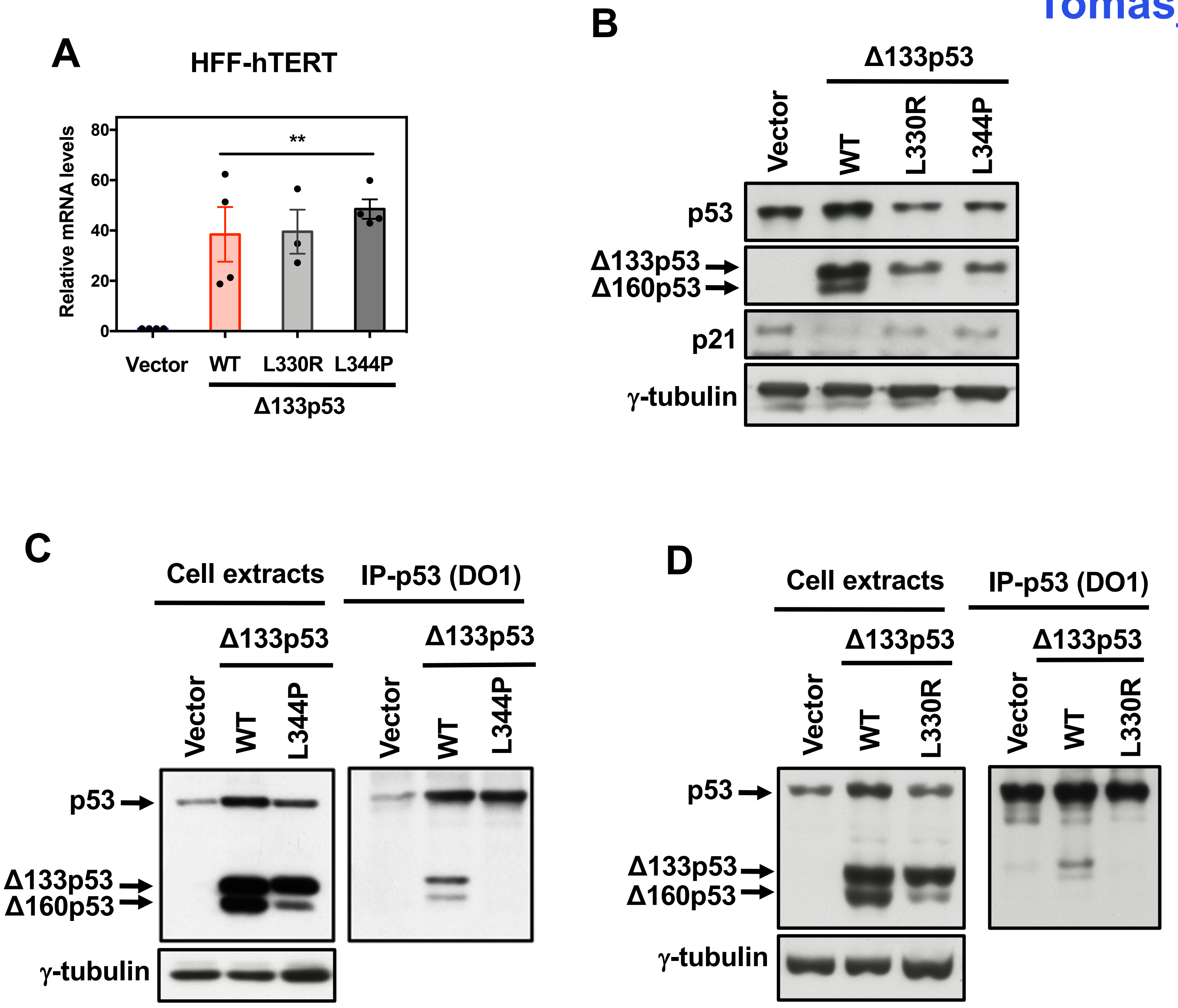

Supplementary Figure S3 (complements Figure 3). Validation of the Δ133p53α-L344P and Δ133p53α-L330R oligomerization mutants

(**A**) Δ133*TP53* mRNA levels were determined by RT-qPCR in HFF-hTERT cells that express Δ133p53α-WT or its oligomerization mutants L344P or L330R. Data are the mean ± SEM (n ≥3); **p <0.01, one-way ANOVA with Dunnett's multiple comparison test.

(**B**) Δ133p53α and Δ160p53α protein levels are lower in HFF-hTERT cells that express the Δ133p53α-L344P or Δ133p53α-L330R oligomeric mutant than in cells that express Δ133p53α-WT (WT). Western blot analysis was performed using the same cell samples described in (**A**) to measure Δ133p53α and Δ160p53α, endogenous p53 and p21 protein levels. Images are representative of three independent experiments.

(**C**) The Δ133p53α-L344P oligomeric mutant cannot form a complex with endogenous p53. Total cell extracts from HFF-hTERT cells that express Δ133p53α-WT (WT), Δ133p53α-L344P or control cells (vector) were immunoprecipitated with the anti-p53 antibody (DO1). Note that the amounts of total extracts from Δ133p53α-L344P expressing cells and Δ133p53α-WT-expressing cells were corrected to have comparable levels of expressed isoforms. Total cell extracts (left panel) and immunoprecipitates (right panel) were immunoblotted with the DO1 and DO11 antibodies. γ-tubulin, loading control. Images are representative of three independent experiments.

(**D**) Same analysis as in (**C**) of cells that express Δ133p53α-L330R. This oligomeric mutant also cannot form a complex with endogenous p53. Images are representative of three independent experiments.

Tomas_Suppl Fig 4

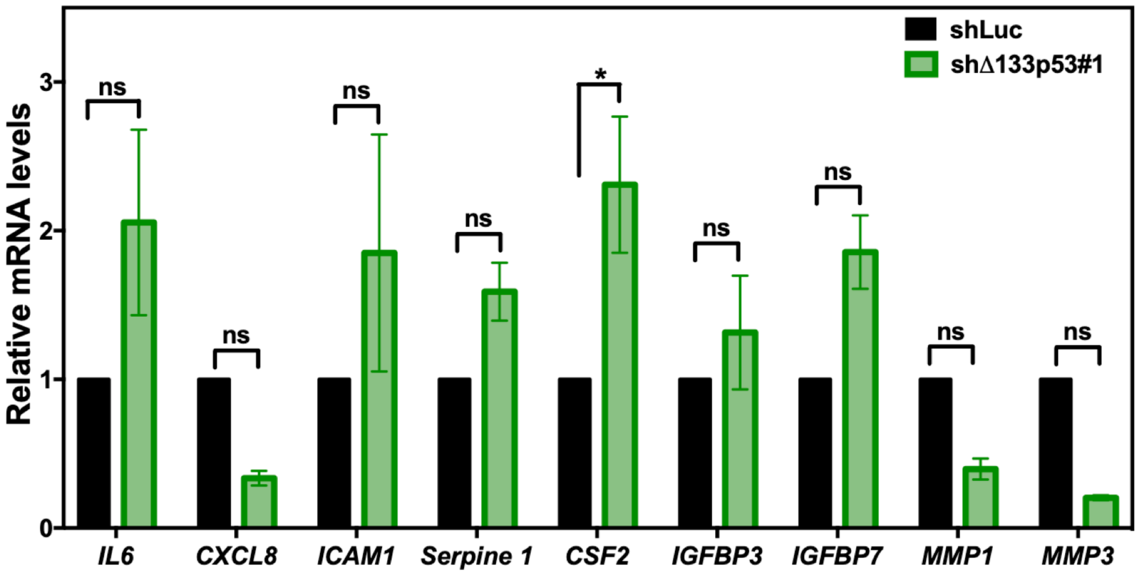

Supplementary Figure S4 (complements Figure 5). Depletion of Δ133*TP53* mRNA does not result in a SASP response

HFF cells were infected with Δ133p53#1 shRNA or sh*Luc* and the expression of the indicated SASP genes was determined by RT-qPCR at day 20 post-infection. Data are the mean ± SEM (n=3); *p <0.05, ns, not significant, two-way ANOVA with Sidak's multiple comparison test.

Tomas_Suppl Fig 5

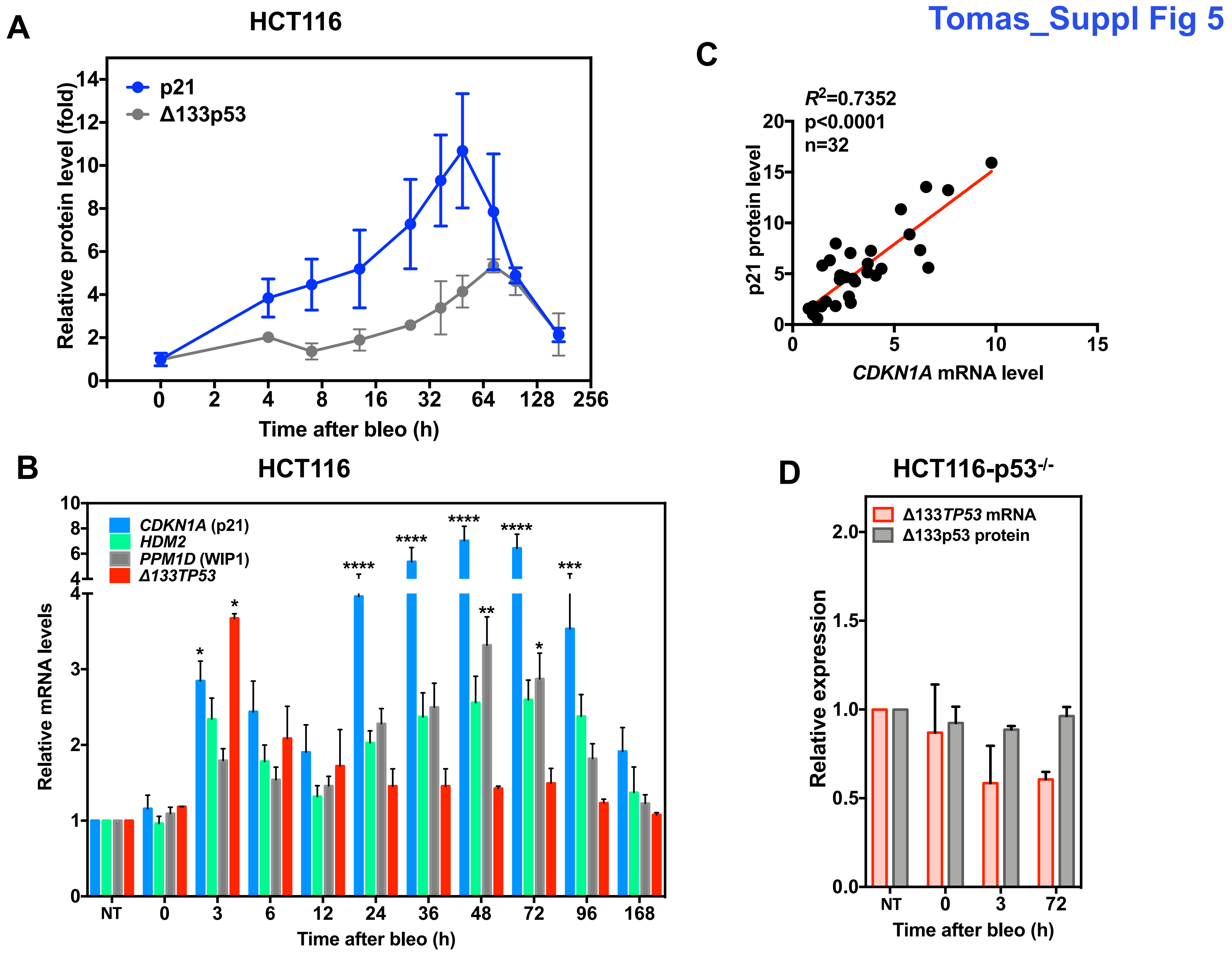

Supplementary Figure S5 (complements Figure 6).

(**A**) HCT116 cells were incubated or not with bleomycin for 1 hour, released and collected at different time points, and p21 and Δ133p53α expression levels were assessed by western blotting as in Fig. 6A. Immunoblots were quantified and p21 and endogenous Δ133p53α levels were normalized to γ-tubulin levels and were relative to the levels in untreated HCT116 cells. Data are the mean ± SEM (n = 3).

(**B**) HCT116 cells were incubated or not with bleomycin for 1 hour, released and collected at different time points, and the relative levels of *CDKN1A*, *HDM2*, *PPM1D* (encoding WIP1) and Δ133*TP53* mRNAs were determined by RT-qPCR. Expression is relative to untreated HCT116 cells. Data are the mean ± SEM (n = 3); *p <0.05, **p <0.01, ***p <0.001, ****p <0.0001, two-way ANOVA with Dunnett's multiple comparison test (vs no treatment; NT).

(**C**) Correlation between p21 protein and *CDKN1A* mRNA levels in the experiment shown in (Fig. 6A and Supplementary Fig. S6A, B). The red line represents the linear regression.

(**D**) HCT116 p53$^{-/-}$ cells were incubated or not with bleomycin for 1 hour, released and collected at 3 and 72 hours after bleomycin treatment. The relative levels of Δ133*TP53* mRNA and protein were determined by RT-qPCR and immunoblotting, respectively. Data are the mean ± SEM (n = 2). NT; no treatment.
