# Supplementary material for: Interaction of p53 with the Δ133p53α and Δ160p53α isoforms regulates p53 conformation and transcriptional activity
Source: Cell Death Dis. 2024 Nov 19;15(11):845. doi: 10.1038/s41419-024-07213-4 (PMC11576908; doi:10.1038/s41419-024-07213-4)
Supplement: Supplementary file 2 — Supplementary Table 1 [file 41419_2024_7213_MOESM2_ESM.pdf]

Antibody	Supplier	Catalog #	Clone name	Application (Dilution)
mouse anti-p53	Santa Cruz	sc-126	DO1	WB (1:250); IF (1:100)
mouse anti-Δ133p53	Bio-Rad	MCA1704	DO11	WB (1:500); IF (1:100)
mouse anti-RB	BD Pharmingen	554136	G3-245	WB (1:250)
rabbit anti-p21	Santa Cruz	sc-397		WB (1:250)
mouse anti-p16	BD Pharmingen	550834	G175-405	WB (1:250)
rabbit anti-p-p53 (Ser 15)	Cell Signaling	9284		WB (1:500)
mouse anti-HDM2	Santa Cruz	sc-965	SMP14	WB (1:250)
mouse anti-WIP1	Santa Cruz	sc-376257	F-10	WB (1:500)
mouse anti–γ-tubulin	Sigma	T6557	GTU-88	WB (1:5000)
rabbit anti-Cyclin A	Santa Cruz	sc-751		WB (1:250)
mouse anti-c-Myc	Hybridoma		9E10	WB (1:50)
rabbit anti-c-Myc	Santa Cruz	sc-789		WB (1:250)
mouse anti-γ-H2AX (Ser139)	Millipore	05-636	JBW301	WB (1:250); IF (1:1000)
goat anti-H2AX	Bethyl Laboratories	A303-837A		WB (1:500)
mouse anti-CHK1	Santa Cruz	sc-56291	DCS-310	WB (1:250)
rabbit anti-p-CHK1 (Ser345)	Cell Signaling	2341		WB (1:1000)
mouse anti-CHK2	Millipore	05-649	clone 7	WB (1:250)
rabbit anti-p-CHK2 (Thr68)	Cell Signaling	2661		WB (1:1000)
rabbit anti-53BP1	Novus Biologicals	NB100-304		IF (1:1000)
mouse anti-BrdU	Bio-Rad	MCA2483GA	Bu20a	IF (1:100)
