# Supplementary material for: Interaction of p53 with the Δ133p53α and Δ160p53α isoforms regulates p53 conformation and transcriptional activity
Source: Cell Death Dis. 2024 Nov 19;15(11):845. doi: 10.1038/s41419-024-07213-4 (PMC11576908; doi:10.1038/s41419-024-07213-4)
Supplement: Supplementary file 3 — Supplementary Table 2 [file 41419_2024_7213_MOESM3_ESM.pdf]

Gene Symbol	Gene description	Primers used for Real-time Q-PCR	Supplier
TP53	tumor protein p53	Forward (5'-3'): TCCATCCAGTGGTTTCTTCTTTG	Eurogentec
(ALL89)		Reverse (5'-3'): GAAGAGAATCTCCGCAAGAAAGG	
		Taqman Probe: AGCACTAAGCGAGCACTGCCCAACA	Applied Biosystem
TP53		Forward (5'-3'): GTGTGGAATCAACCCACAGCT	Eurogentec
(TAP53)		Reverse (5'-3'): CAGCCAAGTCTGTGACTTGCA	
		Taqman Probe: TCCCCTGCCCTCAACAAGATGTTTTGCC	Applied Biosystem
Δ133*TP53*	Δ133p53	Forward (5'-3'): GTGTGGAATCAACCCACAGCT	Eurogentec
		Reverse (5'-3'): ACTCTGTCTCCTTCCTCTTCCTACAG	
		Taqman Probe: TCCCCTGCCCTCAACAAGATGTTTTGCC	
TBP	TATA box binding protein	Forward (5'-3'): CACGAACCACGGCACTGATT	Eurogentec
		Reverse (5'-3'): TTTTCTTGCTGCCAGTCTGGAC	
		Taqman Probe: TGTGCACAGGAGCCAAGAGTGAAGA	Applied Biosystem
BAX	BCL2-associated X protein	QuantiTect Primer Assay Cat# QT00031192	Qiagen
BCL2	B-cell CLL/lymphoma 2	QuantiTect Primer Assay Cat# QT00025011	Qiagen
BIRC5, survivin	baculoviral IAP repeat-containing 5	QuantiTect Primer Assay Cat# QT00081186	Qiagen
BTG2	BTG family, member 2	QuantiTect Primer Assay Cat# QT00240247	Qiagen
C12orf5, Tigar	TP53-induced glycolysis and apoptosis regulator	QuantiTect Primer Assay Cat# QT00071225	Qiagen
CCNA2	Cyclin A2	QuantiTect Primer Assay Cat# QT00014798	Qiagen
CDC25C	cell division cycle 25C	QuantiTect Primer Assay Cat# QT00000350	Qiagen
CDKN1A, p21	cyclin-dependent kinase inhibitor 1A	QuantiTect Primer Assay Cat# QT00062090	Qiagen
CDKN2A, p16	cyclin-dependent kinase inhibitor 2A	QuantiTect Primer Assay Cat# QT00089964	Qiagen
CSF2	colony stimulating factor 2	QuantiTect Primer Assay Cat# QT00000896	Qiagen
CXCL8	C-X-C motif chemokine ligand 8	QuantiTect Primer Assay Cat# QT00000322	Qiagen
DRAM1	DNA damage regulated autophagy modulator 1	QuantiTect Primer Assay Cat# QT00098350	Qiagen
SESN1	sestrin 1	QuantiTect Primer Assay Cat# QT0001133	Qiagen
DRAM1	DNA damage regulated autophagy modulator 1	QuantiTect Primer Assay Cat# QT00098350	Qiagen
GADD45A	growth arrest and DNA-damage-inducible, alpha	QuantiTect Primer Assay Cat# QT00014084	Qiagen
GDF15	growth differentiation factor 15	QuantiTect Primer Assay Cat# QT00082558	Qiagen
HDM2	HDM2 proto-oncogene, E3 ubiquitin protein ligase	QuantiTect Primer Assay Cat# QT00056378	Qiagen
ICAM-1	intercellular adhesion molecule 1	QuantiTect Primer Assay Cat# QT00074900	Qiagen
IGFBP3	insulin-like growth factor binding protein 3	QuantiTect Primer Assay Cat# QT00072737	Qiagen
IGFBP7	insulin-like growth factor binding protein 7	QuantiTect Primer Assay Cat# QT00076720	Qiagen
IL6	interleukin-6	QuantiTect Primer Assay Cat# QT00083720	Qiagen
MMP1	matrix metallopeptidase 1	QuantiTect Primer Assay Cat# QT00014581	Qiagen
MMP3	matrix metallopeptidase 3	QuantiTect Primer Assay Cat# QT00060025	Qiagen
PMAIP1, Noxa	PMA-induced protein 1	QuantiTect Primer Assay Cat# QT01006138	Qiagen
PPM1D	protein phosphatase, Mg2+/Mn2+ dependent, 1D	QuantiTect Primer Assay Cat# QT00033425	Qiagen
PTP4A1	protein tyrosine phosphatase 4A1	QuantiTect Primer Assay Cat# QT00028203	Qiagen
Serpine 1, PAI-1	plasminogen activator inhibitor 1	QuantiTect Primer Assay Cat# QT00062496	Qiagen
SESN1	sestrin 1	QuantiTect Primer Assay Cat# QT0001133	Qiagen
TP53I3, PIG3	tumor protein p53 inducible protein 3	QuantiTect Primer Assay Cat# QT00010332	Qiagen
