# Supplementary material for: Interaction of p53 with the Δ133p53α and Δ160p53α isoforms regulates p53 conformation and transcriptional activity
Source: Cell Death Dis. 2024 Nov 19;15(11):845. doi: 10.1038/s41419-024-07213-4 (PMC11576908; doi:10.1038/s41419-024-07213-4)

Tomas Figure 1A

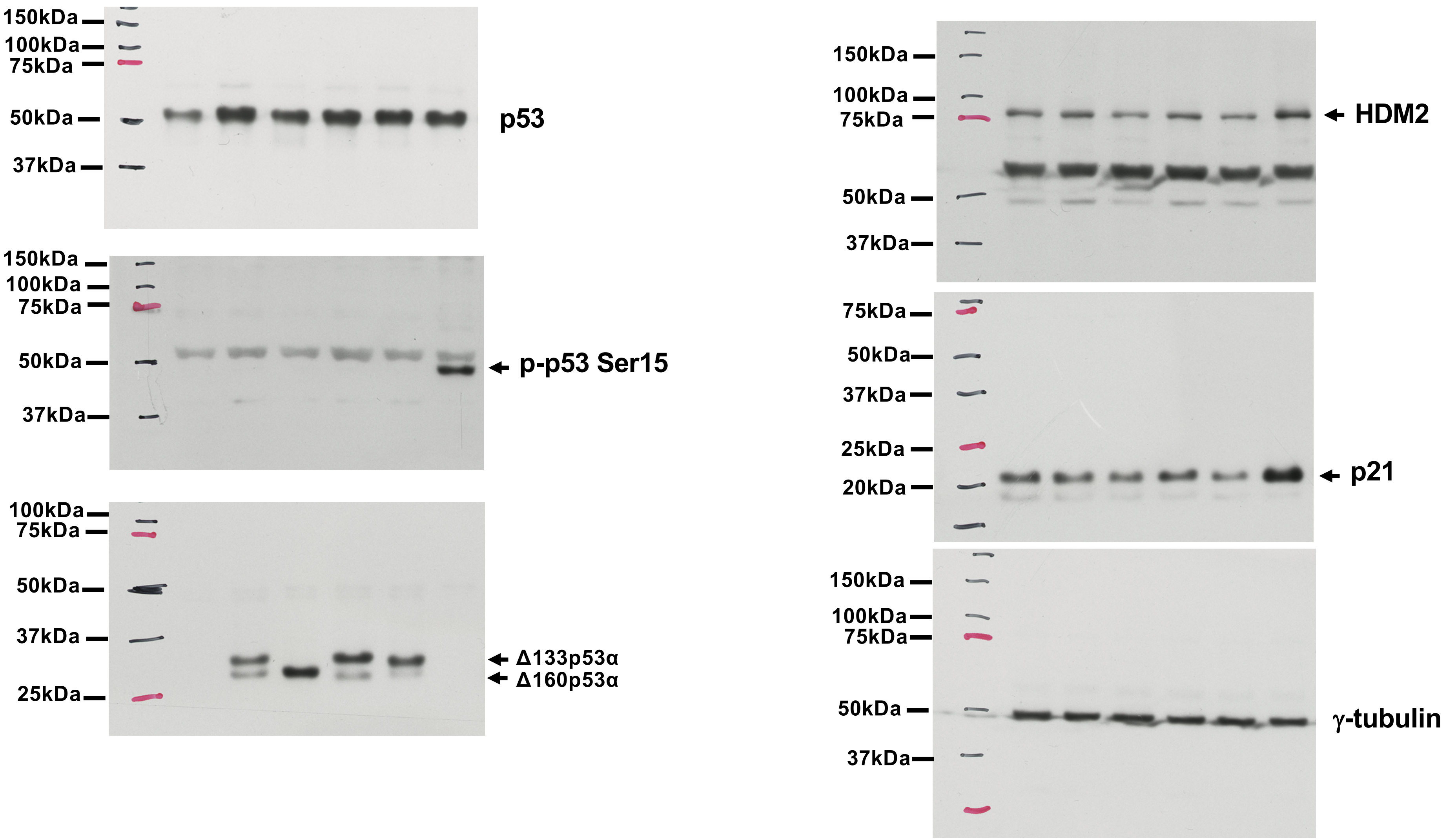

Tomas Figure 1D

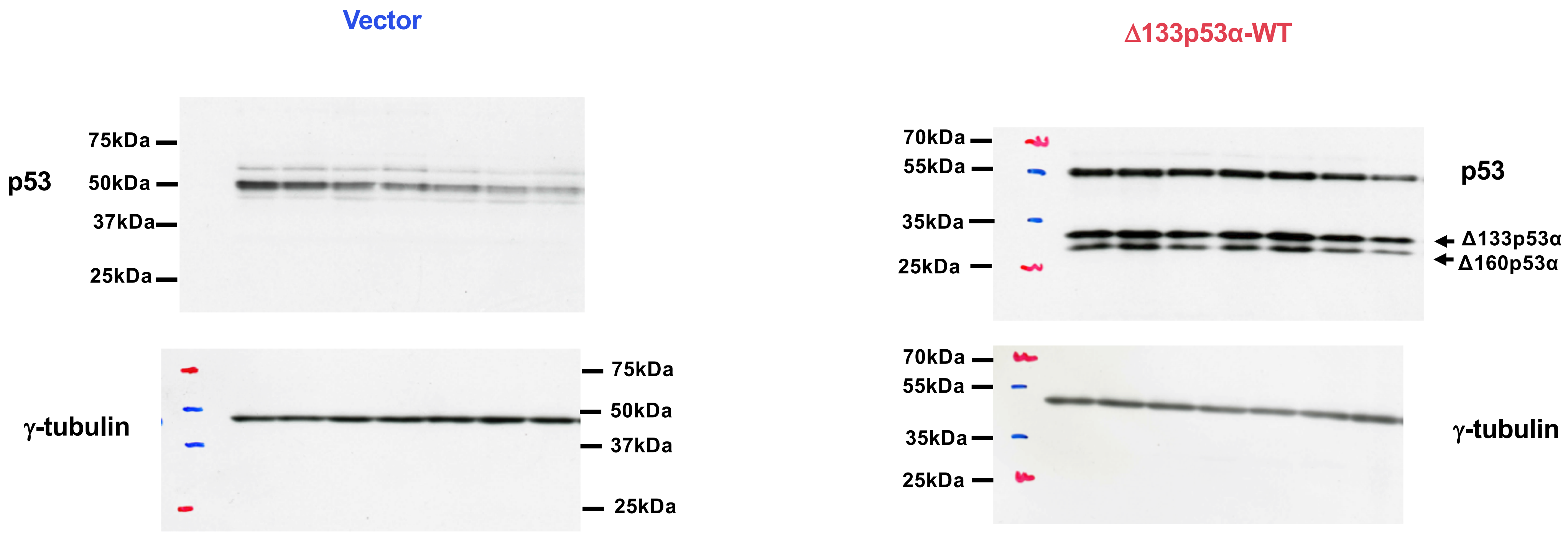

Tomas Figure 1 E

(Note: The membrane from this experiment was incubated first in **DO1** then in **DO11** and then in **γ-tubulin** (traces of **DO1 and DO11** can be seen on the **γ-tubulin** image).

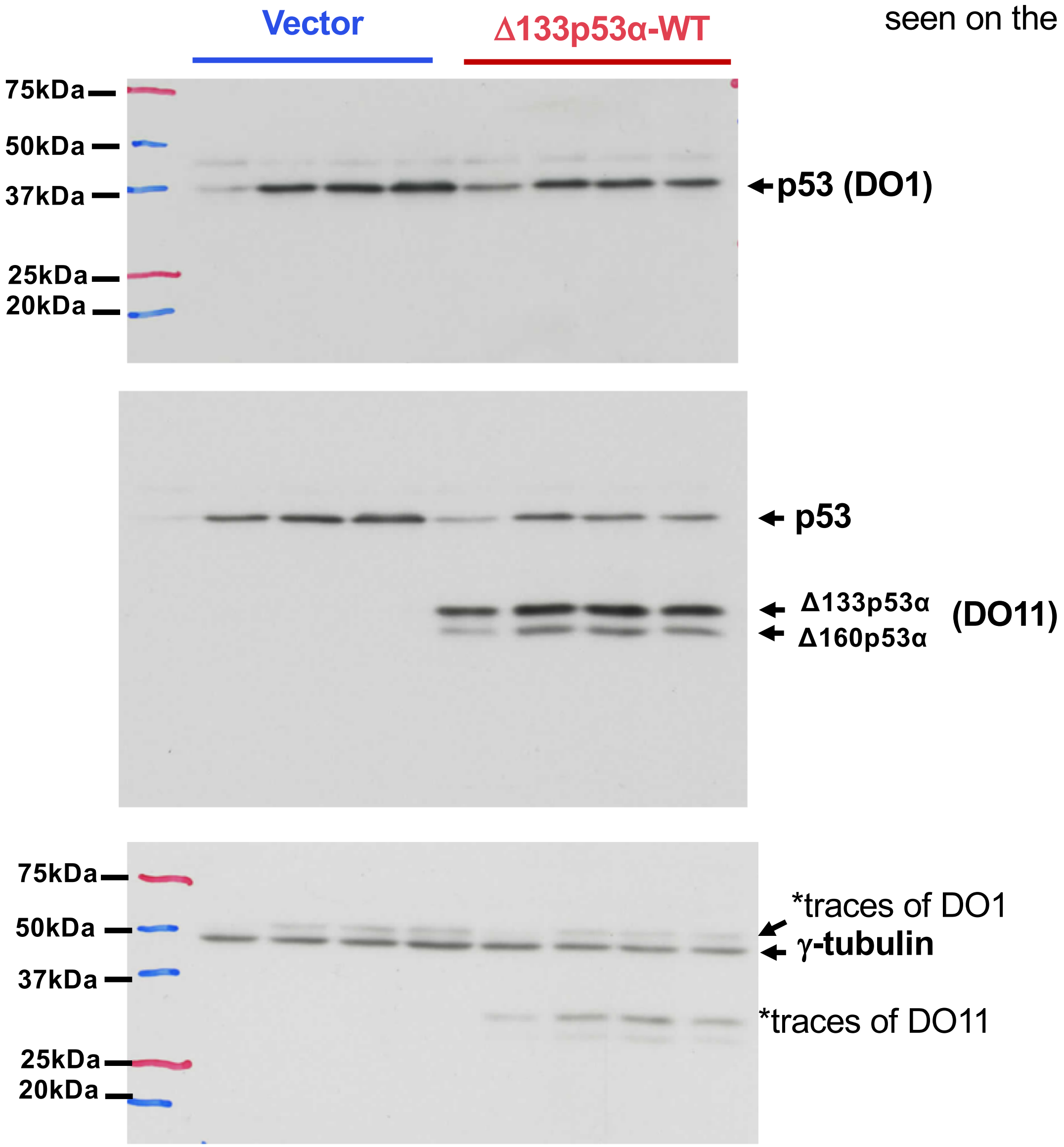

Tomas Figure 2 A

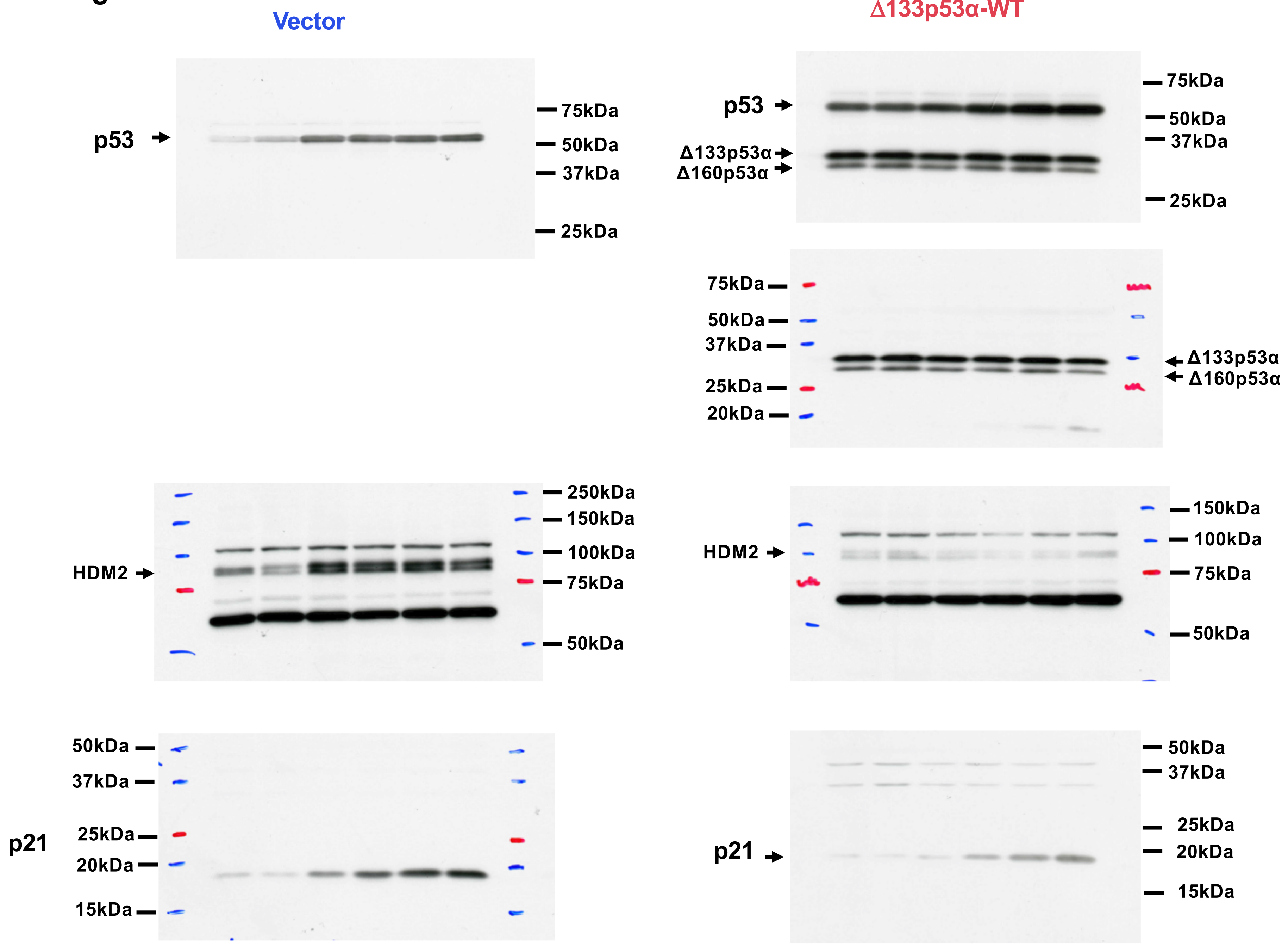

Tomas Figure 2 A

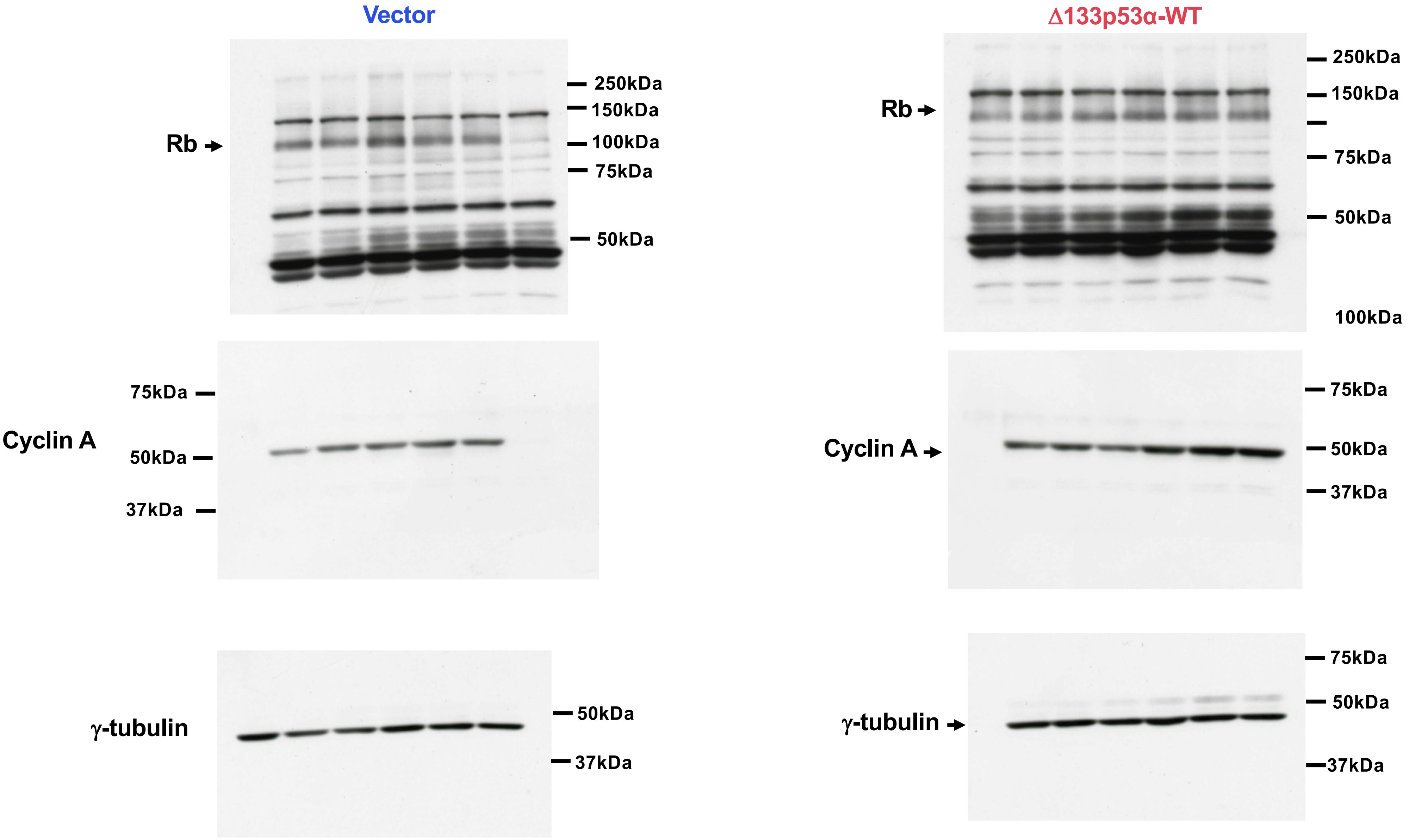

Tomas Figure 3 B

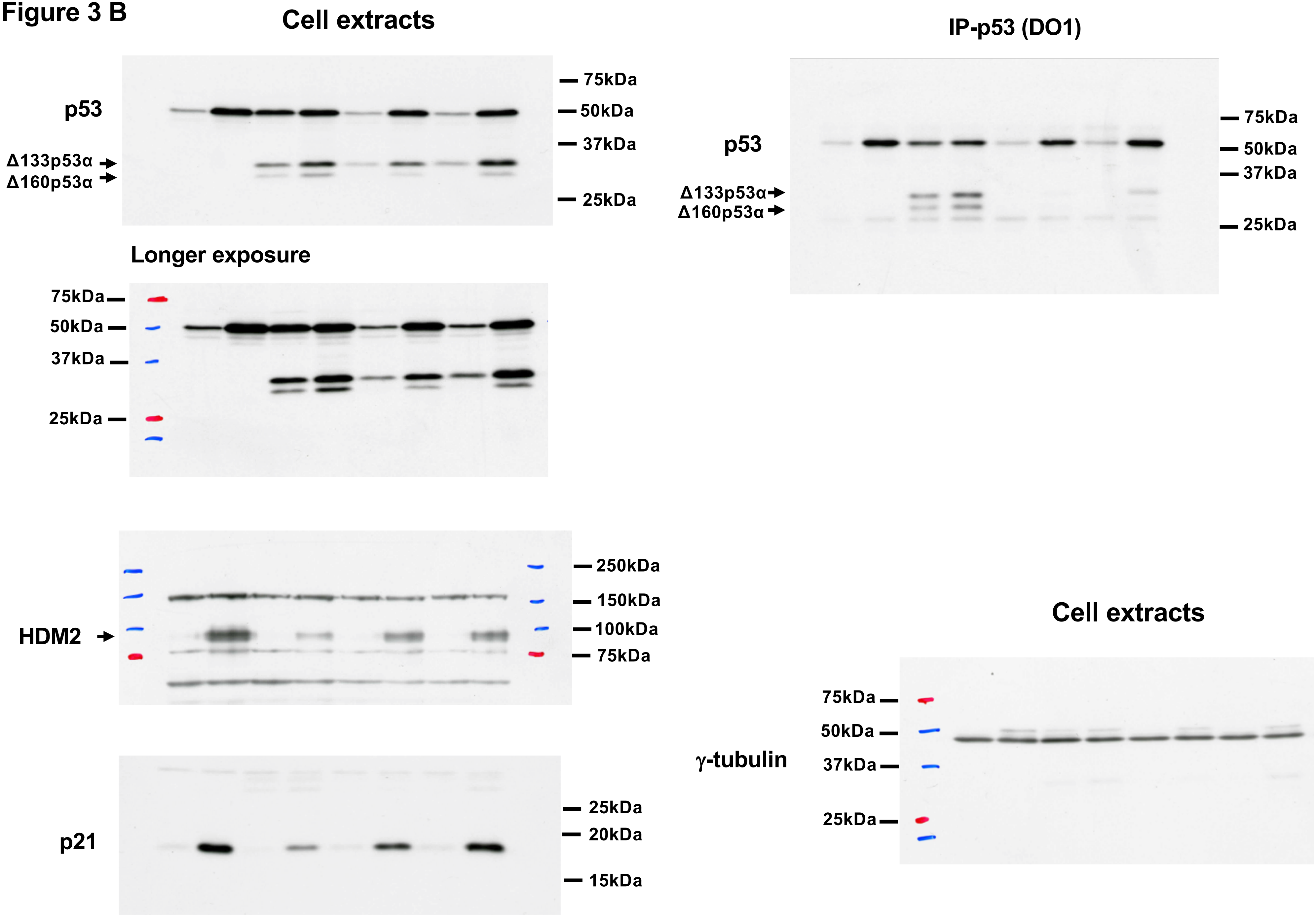

Tomas Figure 4A

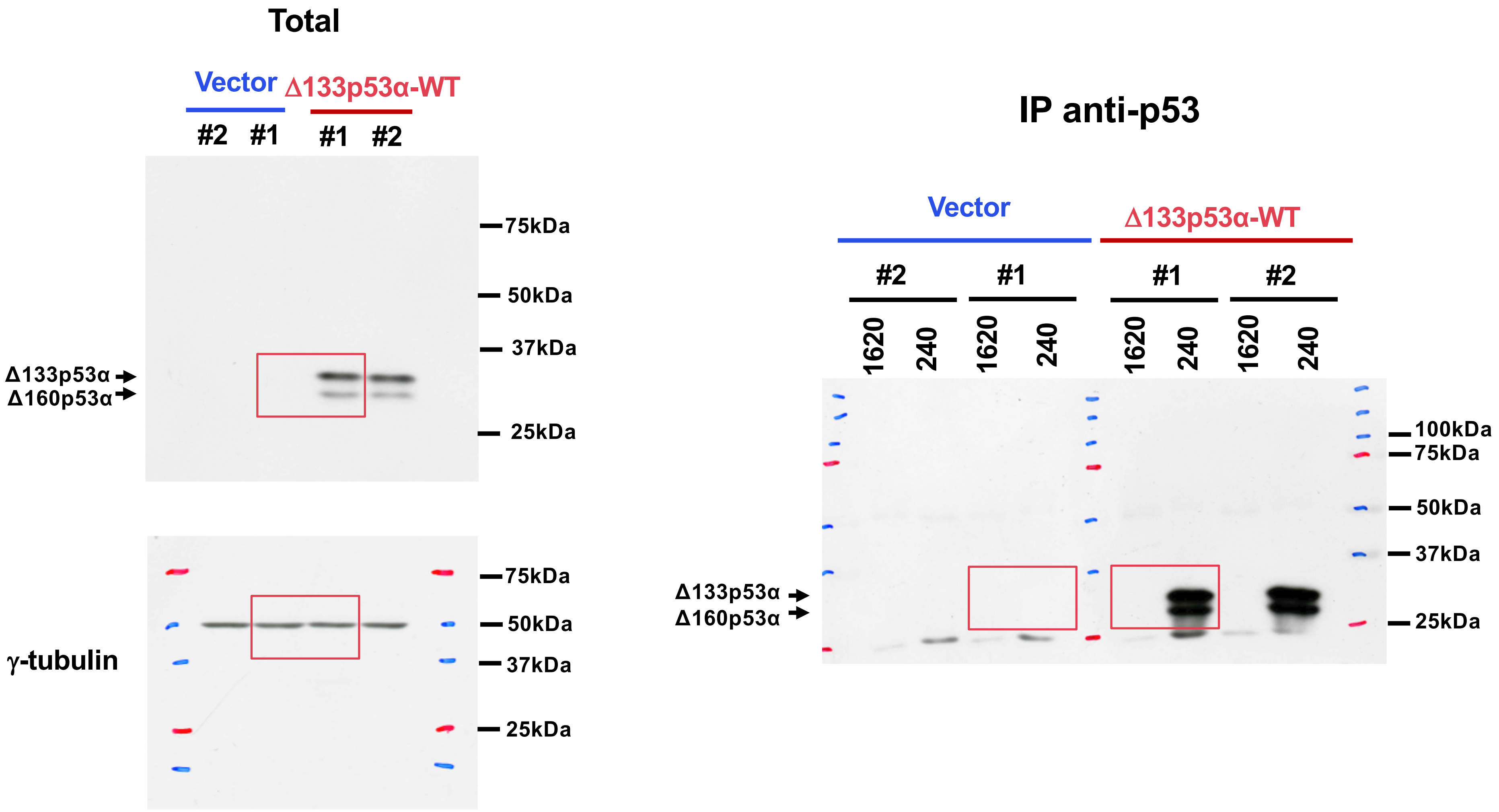

The rectangle indicates biological replicate#1 in Figure 4A

Tomas Figure 4 B

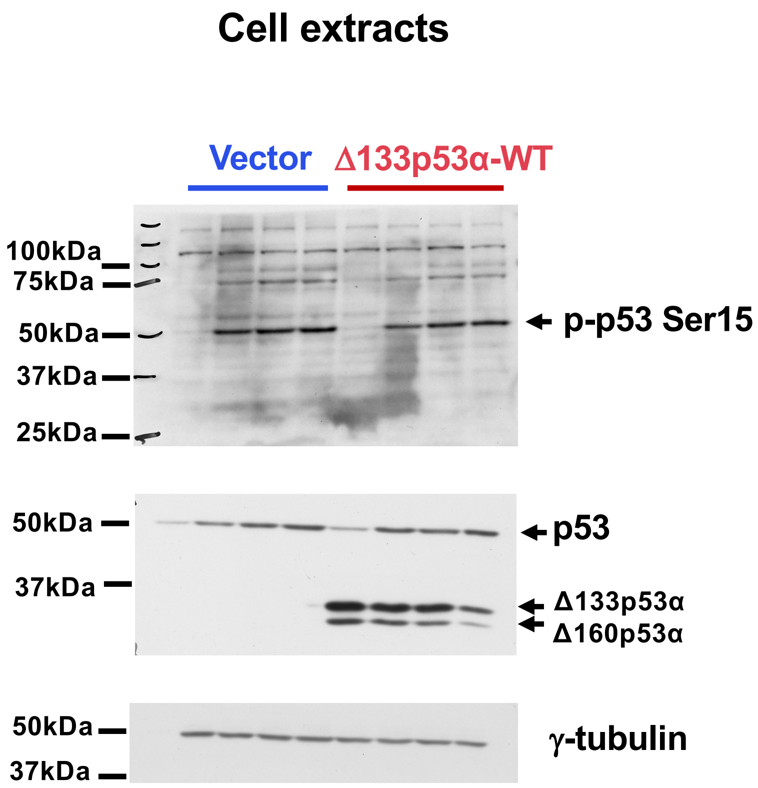

Tomas Figure 4 C

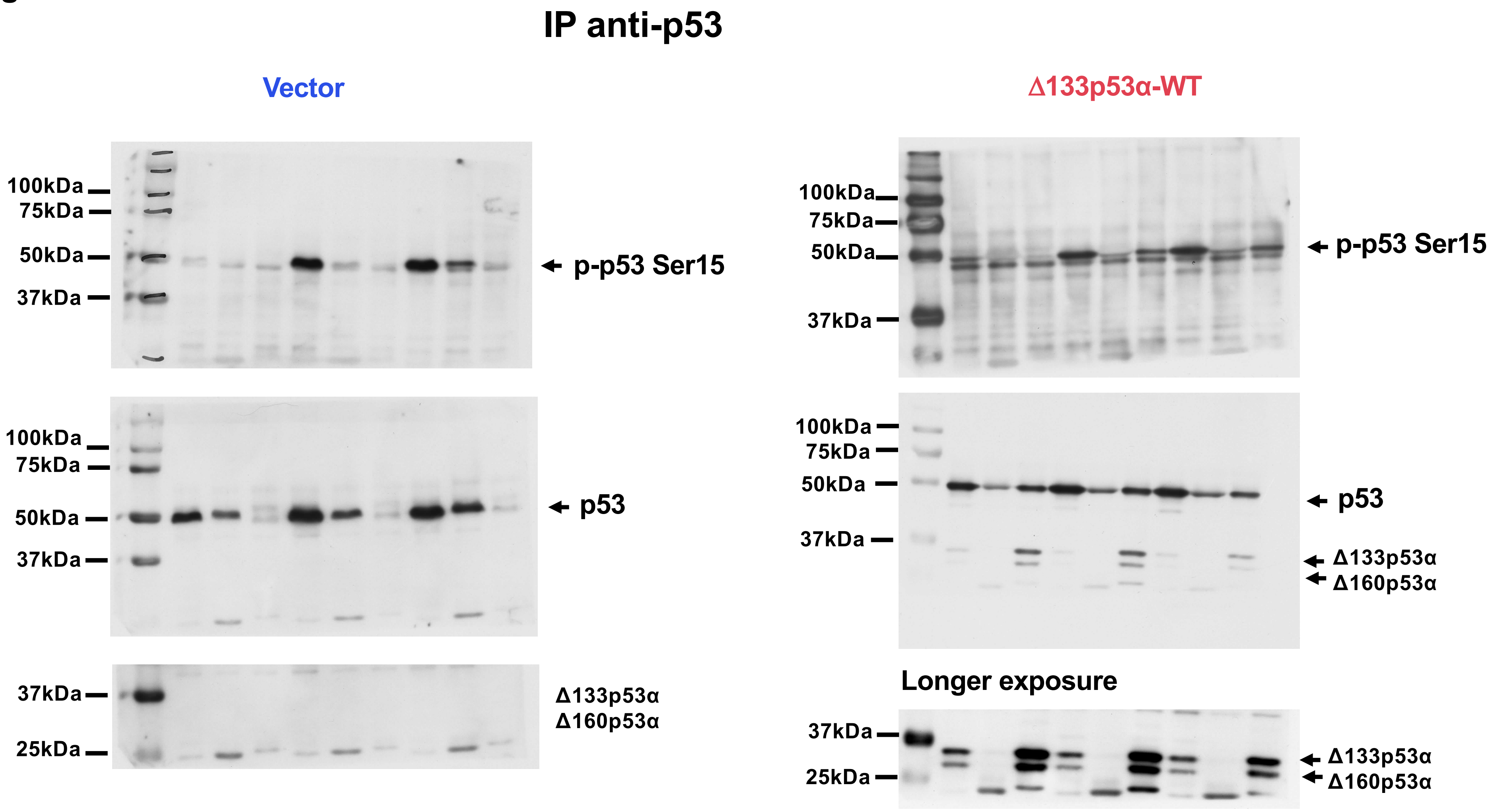

Tomas Figure 5F

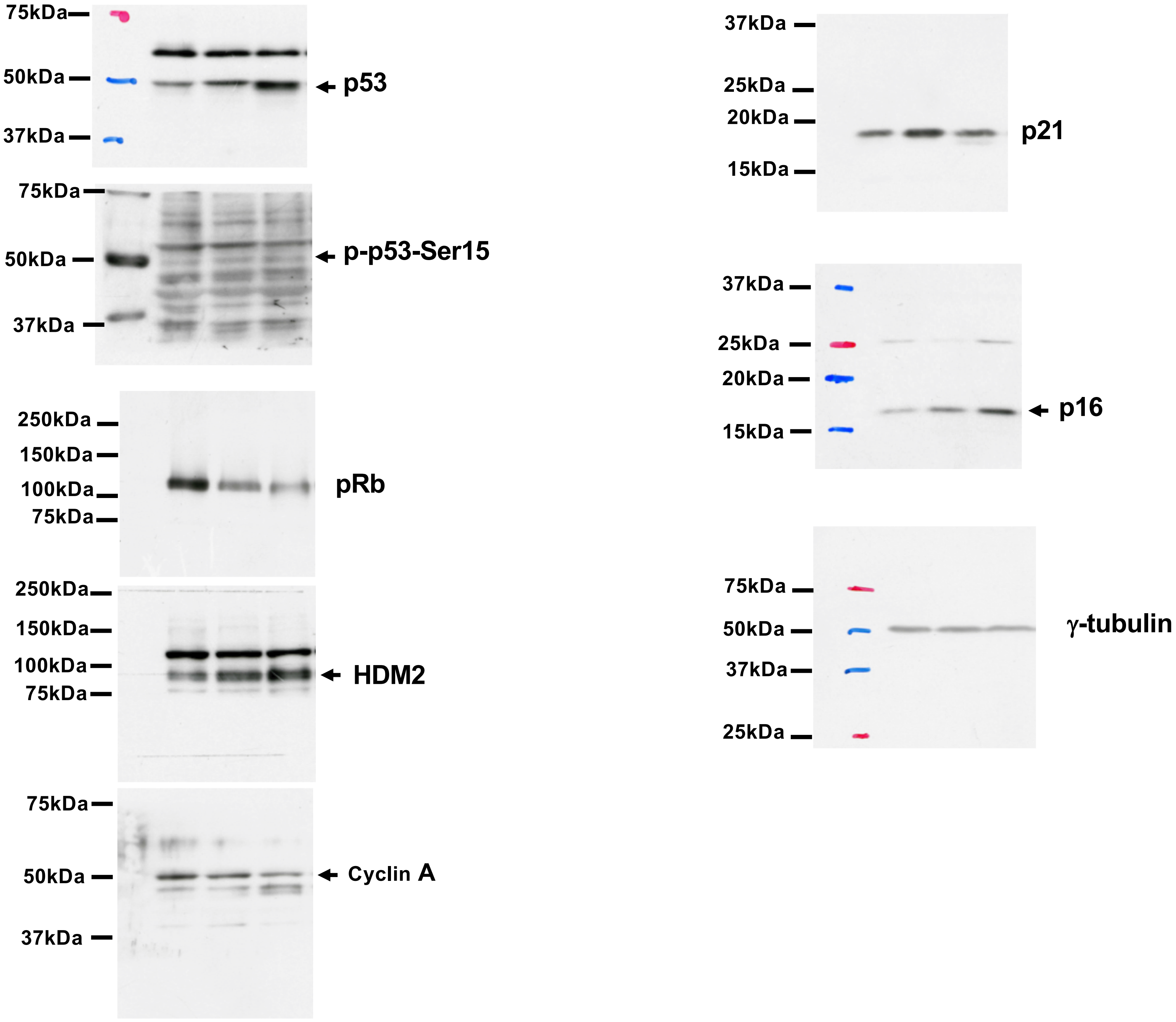

Tomas Figure 6A **Cell extracts**

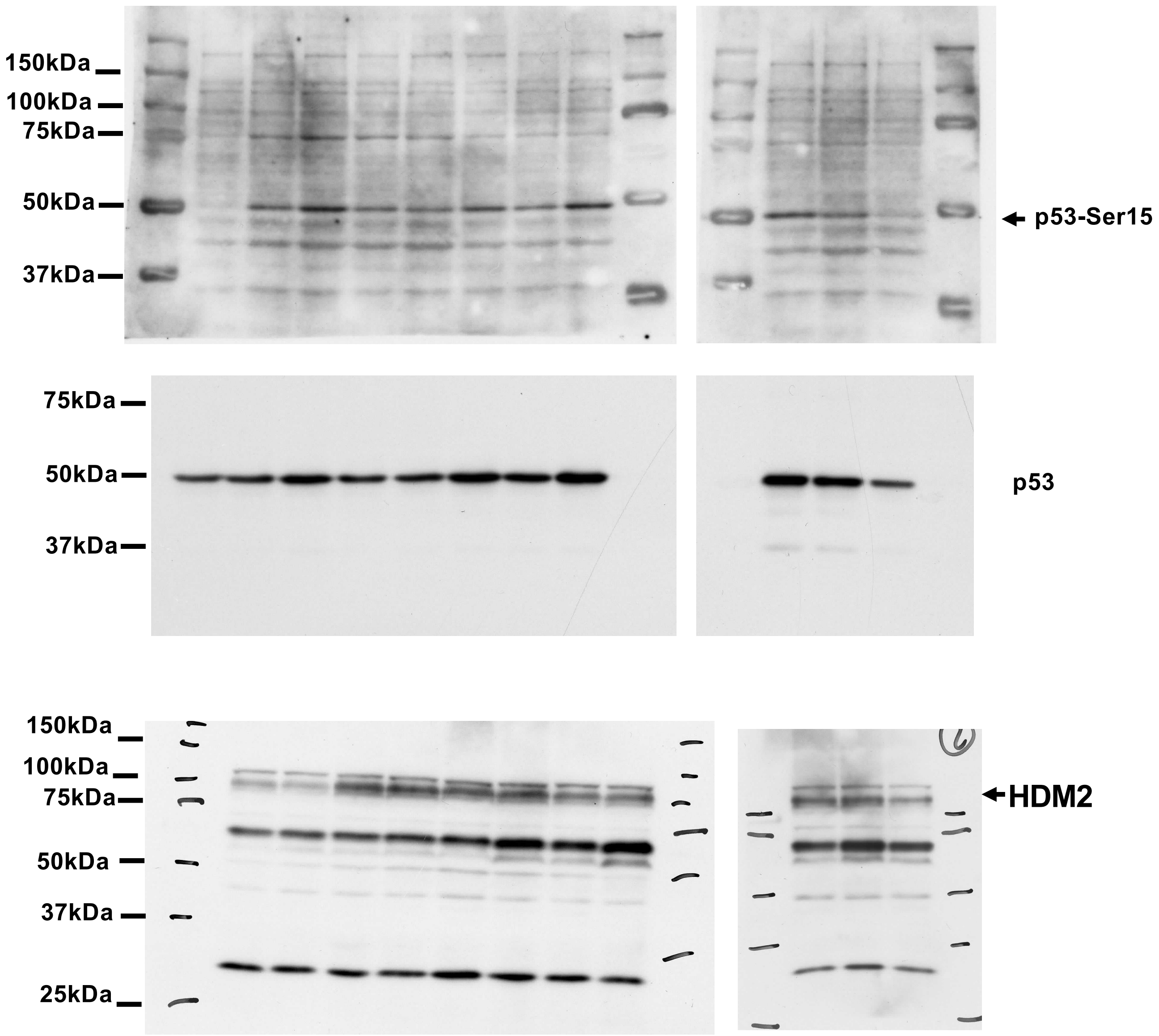

Tomas Figure 6A

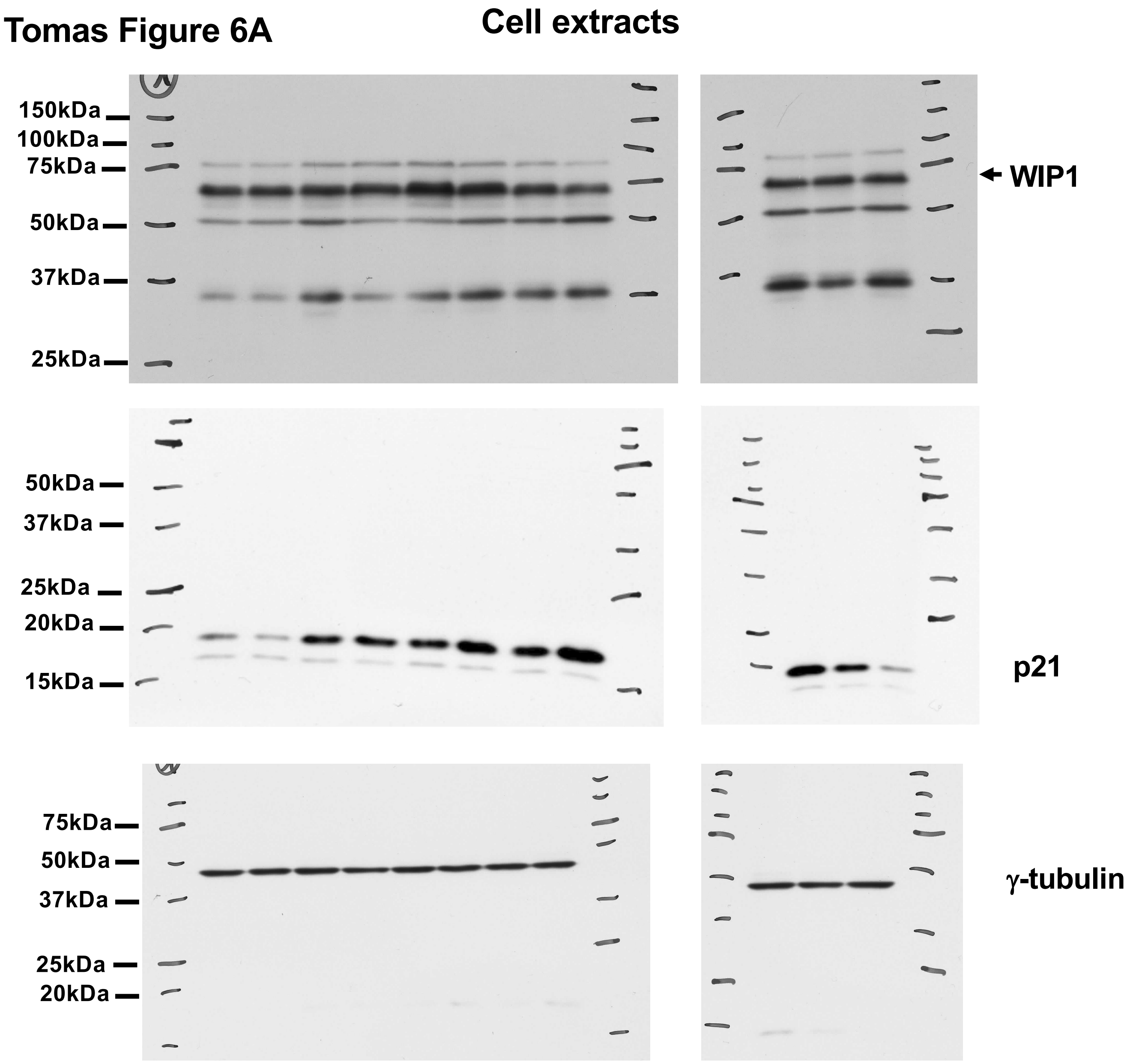

Tomas Figure 6E

Cell extracts

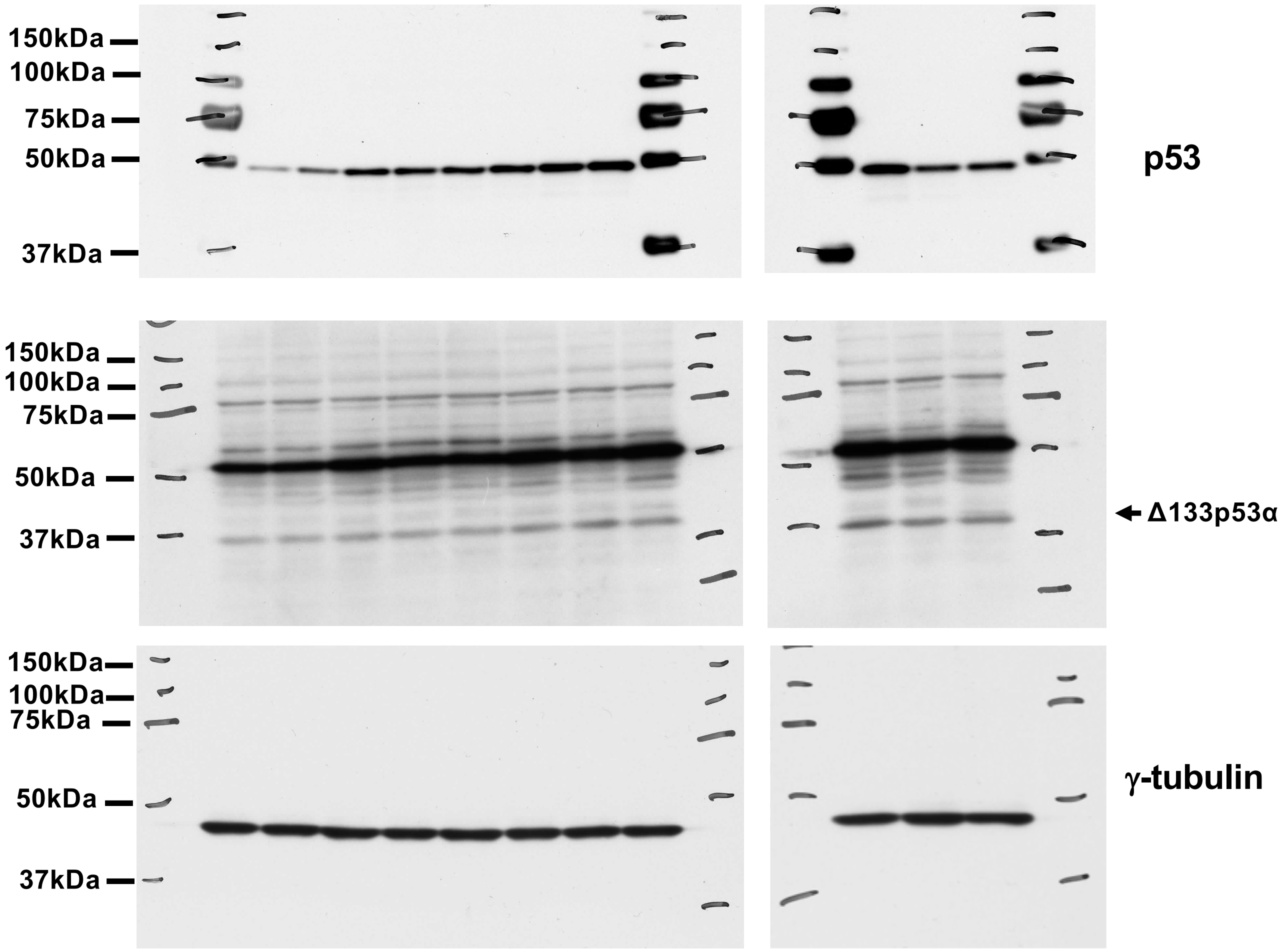

Tomas Figure 6E

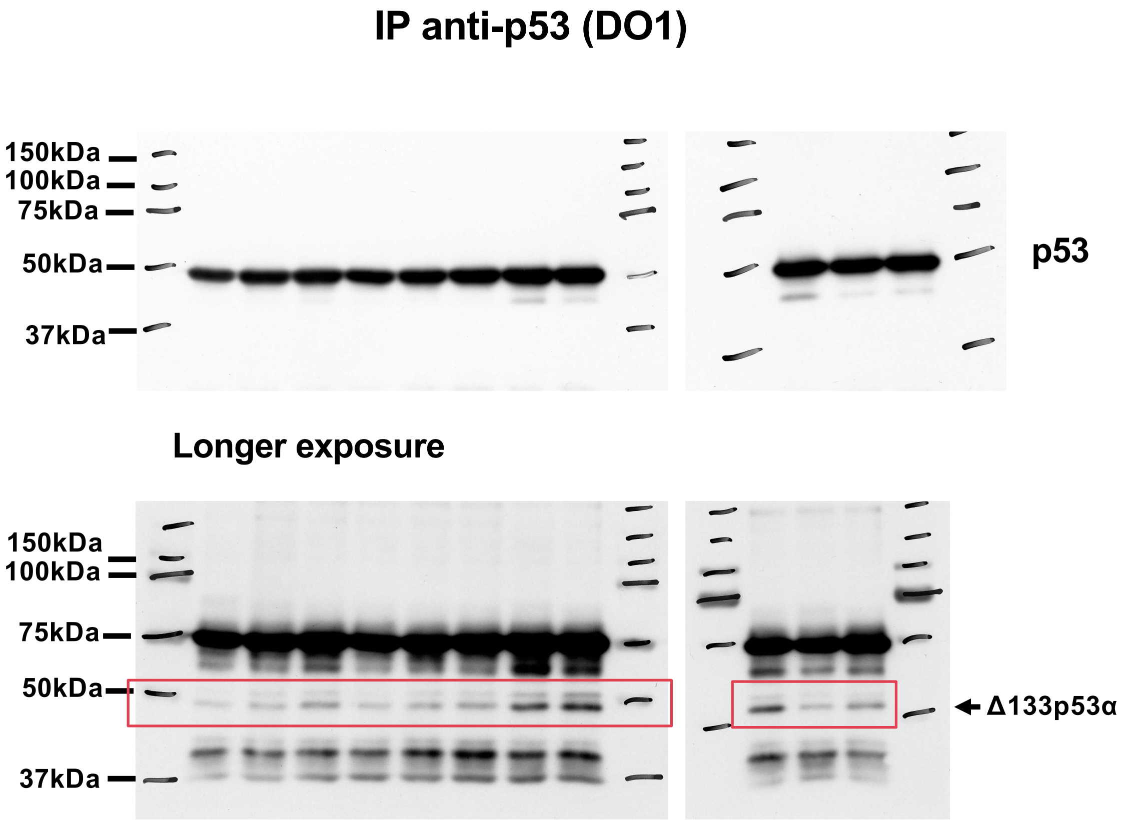

Tomas_Suppl Figue 1A

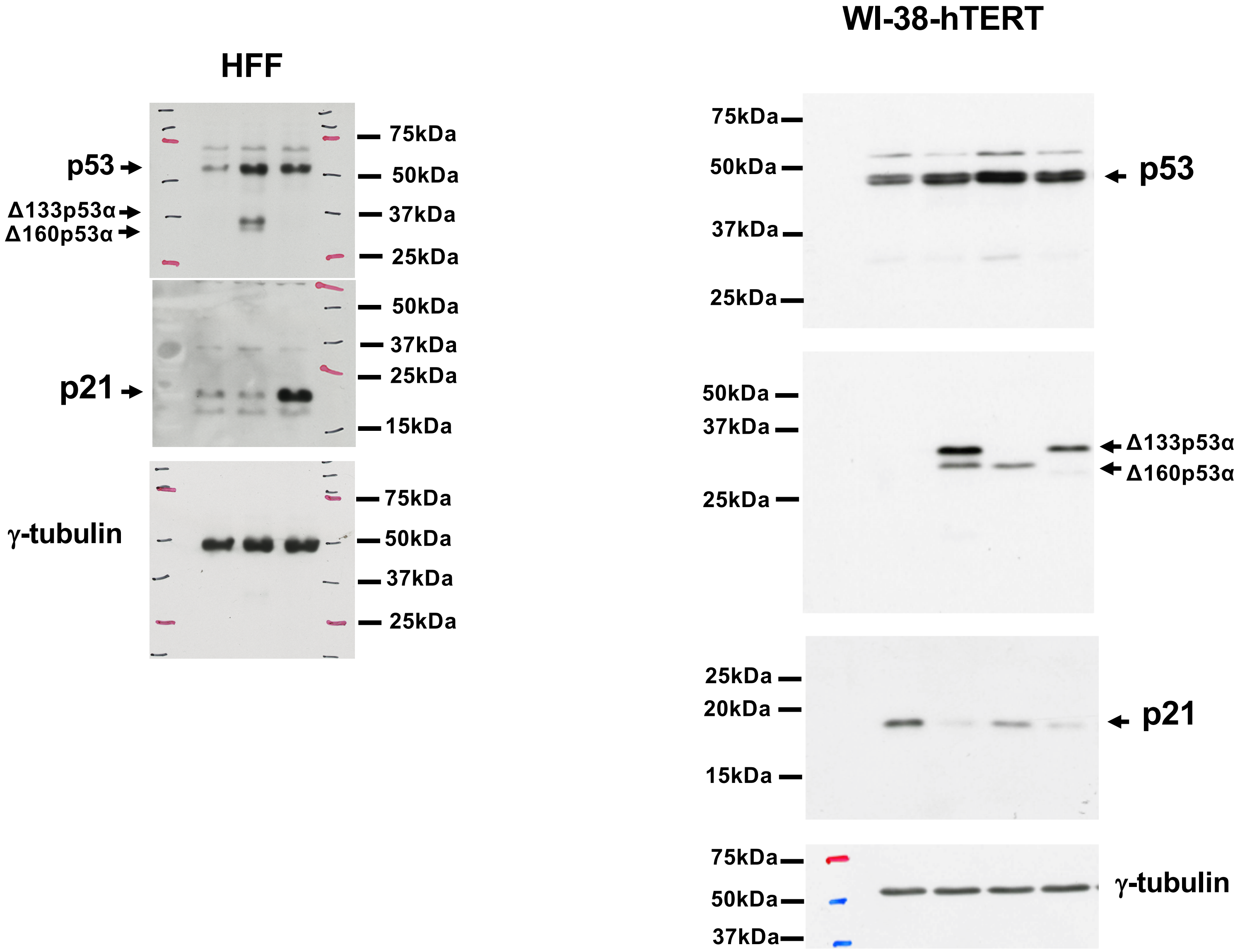

Tomas_Suppl Figue 1C

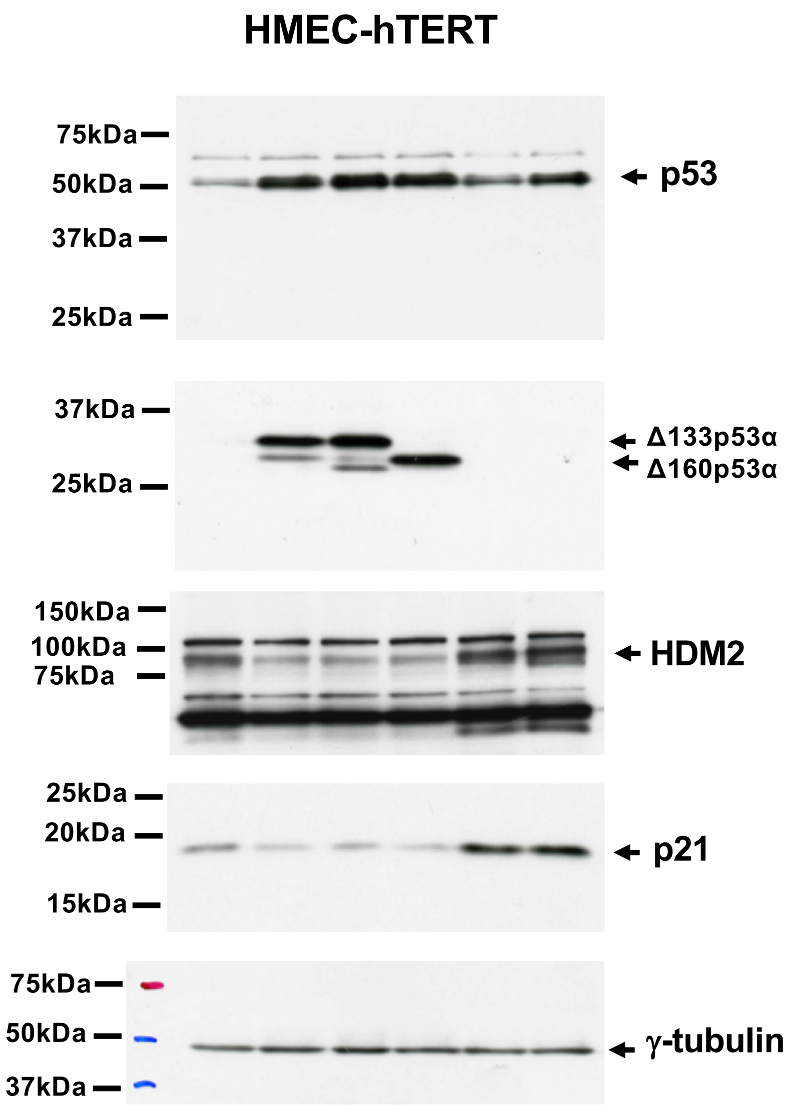

Tomas_Suppl Figure 2 A

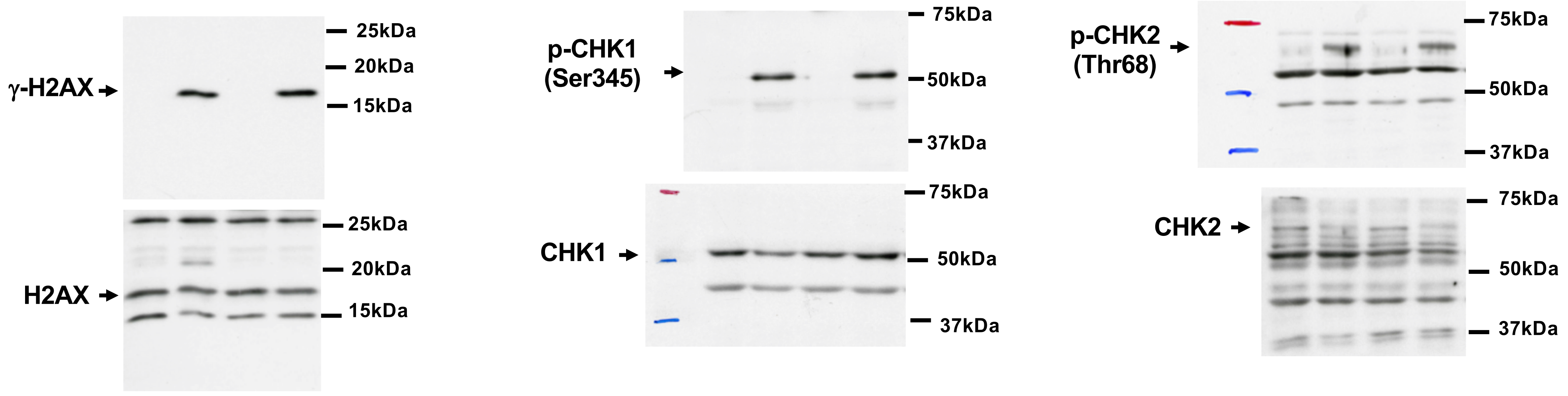

(Note: This membrane was first incubated with p-CHK2 and then with p-p53-ser15, which is why there are traces of p-CHK2 on the p-p53-ser15 image).

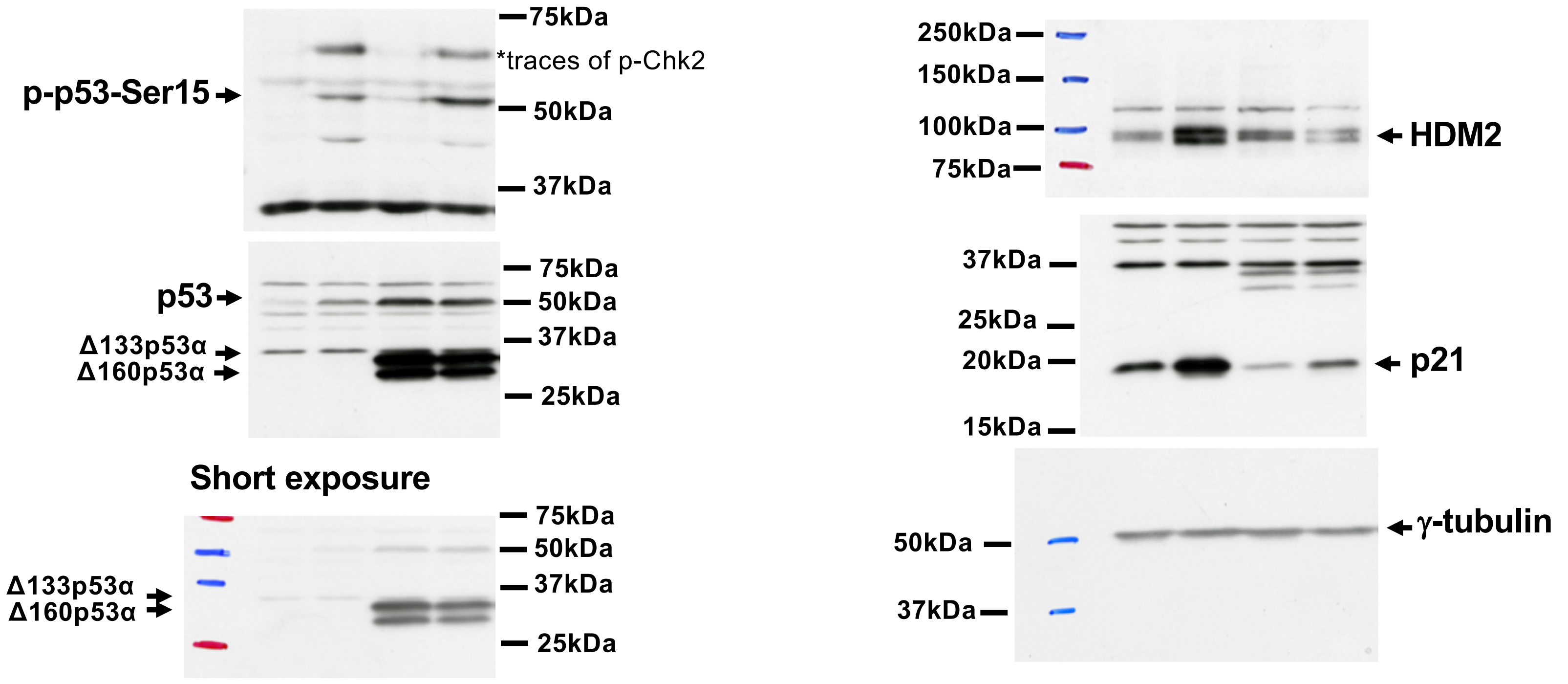

Tomas_Suppl Figure 3 B

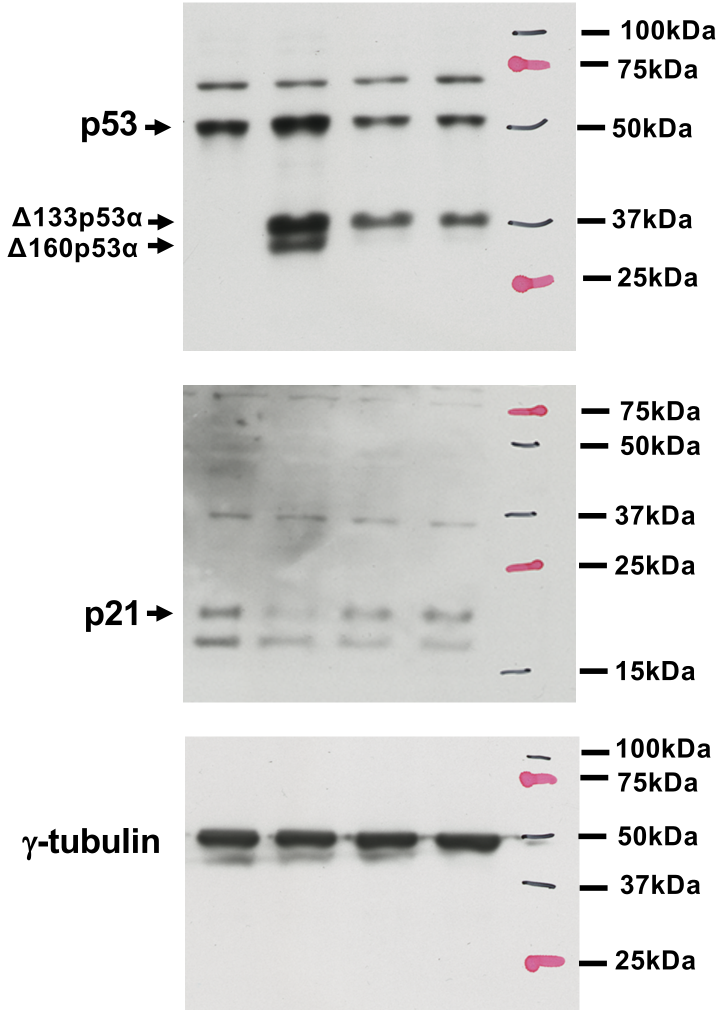

Tomas_Suppl Figure 3 C

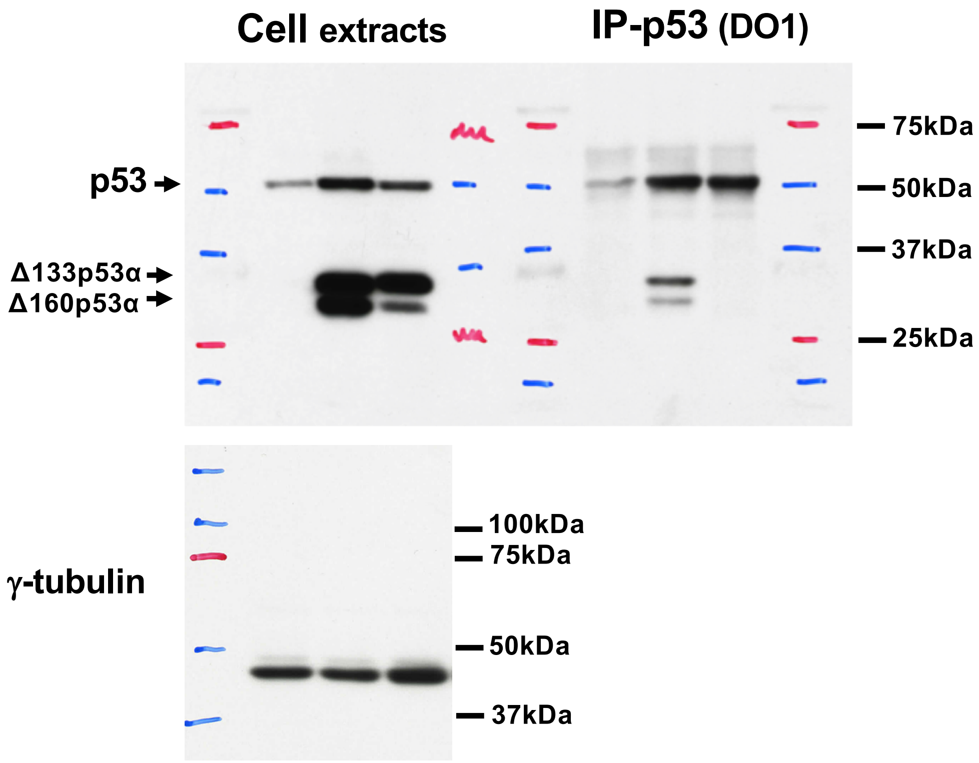

Tomas_Suppl Figure 3 D

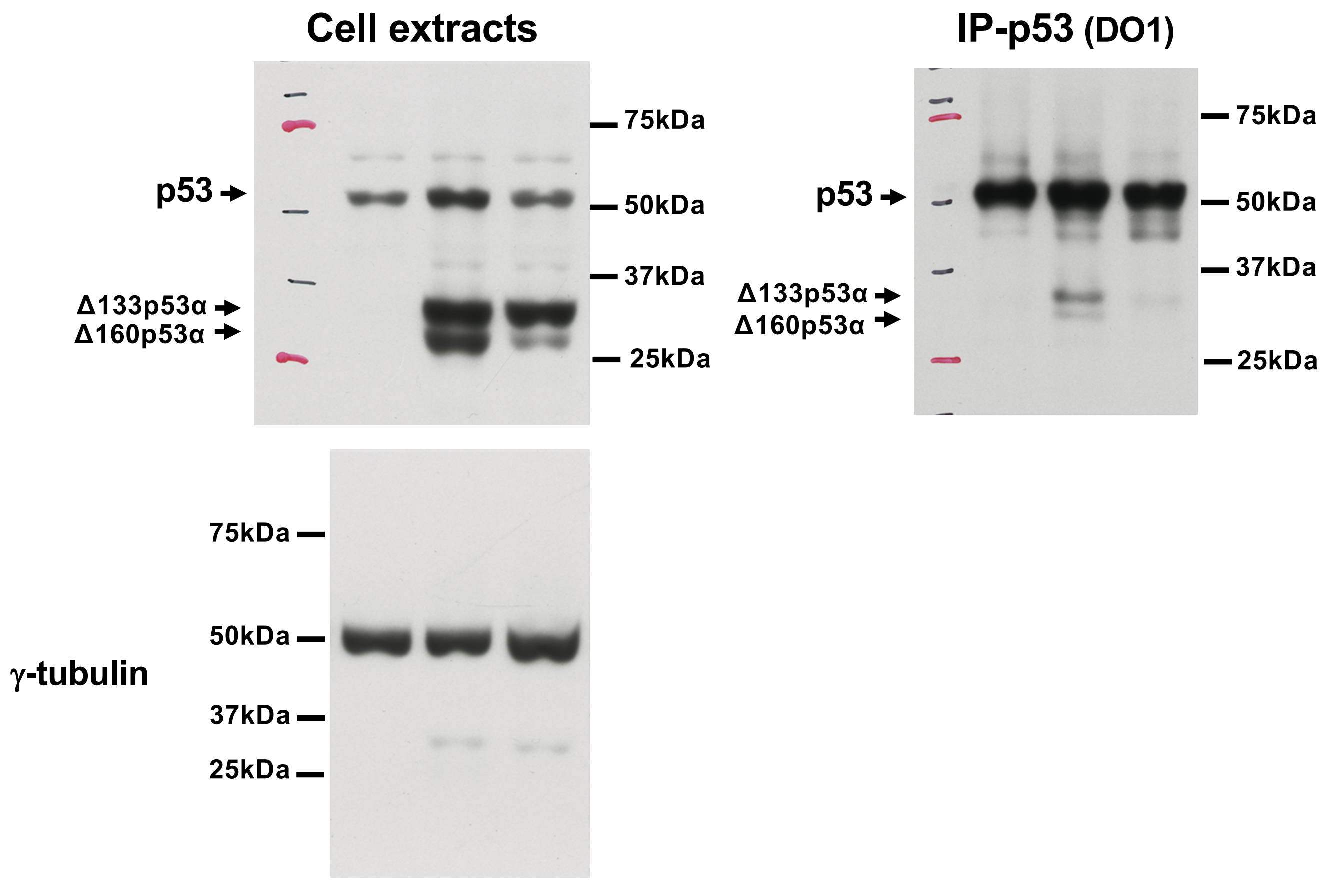

Supplement: Supplementary file 4 — uncropped original Western Blots [file 41419_2024_7213_MOESM4_ESM.pdf]
